# Temporal and Partial Reversal of Airflow Limitation in Patients With COPD Treated With Single‐Inhaler Long‐Acting Dual Bronchodilators

**DOI:** 10.1111/crj.70173

**Published:** 2026-04-20

**Authors:** Yimeng Lu, Yanan Zhou, Liping Xue, Hao Tian, Junjie Han, Yanping Yang, Yunxin Guo, Weipeng Jiang, Cuicui Chen, Linlin Wang, Yuanlin Song

**Affiliations:** ^1^ Department of Pulmonary and Critical Care Medicine, Zhongshan Hospital, Shanghai Medical College Fudan University Shanghai China; ^2^ School of Information Science and Technology Fudan University Shanghai China; ^3^ Shanghai Key Laboratory of Lung Inflammation and Injury, Department of Pulmonary Medicine, Zhongshan Hospital Fudan University Shanghai China; ^4^ Shanghai Institute of Infectious Disease and Biosecurity Shanghai China; ^5^ Shanghai Respiratory Research Institute Shanghai China; ^6^ National and Shanghai Clinical Research Center for Aging and Medicine, Huashan Hospital Fudan University Shanghai China

**Keywords:** chronic obstructive pulmonary disease (COPD), dual bronchodilators, lung function, reversal of airflow limitation

## Abstract

**Introduction:**

Chronic obstructive pulmonary disease (COPD) is characterized by irreversible airflow limitation. While single‐inhaler long‐acting dual bronchodilators (LADBs) effectively relieve symptoms and improve lung function, their long‐term impact on spirometry outcomes and the potential for meaningful changes in key diagnostic indices in a real‐world setting remain underexplored.

**Methods:**

This was a single‐country (China), single‐center, retrospective, noninterventional, real‐world study that included 182 patients treated with LADBs (indacaterol/glycopyrronium [IND/GLY] or umeclidinium/vilanterol [UMEC/VI]). The primary outcomes were changes in trough FEV1 and FEV1/FVC from baseline. Secondary outcomes were changes in other spirometry parameters. An exploratory post hoc analysis explored the reversal of FEV1/FVC, defined as a posttreatment trough FEV1/FVC ratio that exceeded 70%.

**Results:**

Single‐inhaler LADB therapy significantly improved lung function, including FEV1, FVC, FEV1/FVC, RV, and parameters of small airway function. The least‐square mean change in trough FEV1 and FEV1/FVC post‐treatment was 0.154 L [95% CI: 0.09, 0.218] and 1.929% [95% CI: 0.694, 3.164], respectively, at 24 weeks. Posttreatment FEV1 exhibited a characteristic pattern: an initial increase, followed by a peak, a subsequent slight decline, and eventual stabilization. Patients with lower GOLD grades experienced greater improvement in key spirometry parameters. Notably, 21 patients (11.5%) achieved a reversal of the FEV1/FVC ratio to over 70%. Exploratory logistic regression showed that a better baseline lung function, especially FEV1/FVC ratio, was associated with an increased likelihood of achieving this threshold (OR = 1.52 [95% CI: 1.29, 1.89]).

**Conclusion:**

This study demonstrated significant and sustained improvements in lung function in this real‐world cohort of COPD patients managed with LABA/LAMA monotherapy. A subset of patients achieved a posttreatment FEV1/FVC ratio above 70%, indicating a potentially meaningful change in airflow limitation after LADB therapy.

## Graphical abstract

Single‐inhaler dual bronchodilator therapy significantly improved FEV1, FEV1/FVC, and other spirometry parameters in patients with COPD. The improvements in FEV1 and FEV1/FVC exhibits different trends. Notably, 21 patients (11.5%) achieved a reversal of the FEV1/FVC ratio to over 70%. Better baseline lung function was associated with a higher likelihood of FEV1/FVC reversal.

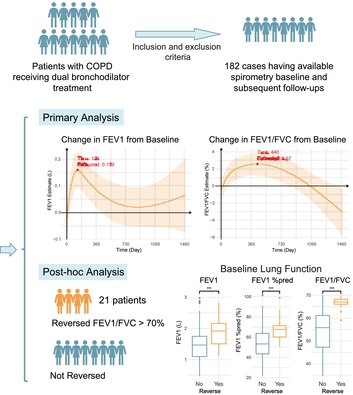

**Abbreviations**
BDR: bronchodilator response
CI: confidence interval
COPD: chronic obstructive pulmonary disease
DLCO/VA: diffusing capacity per unit alveolar volume
FEF: forced expiratory flow
FeNO: fractional exhaled nitric oxide
FEV1: forced expiratory volume in 1 s
FEV1/FVC: ratio of forced expiratory volume in 1 s to forced vital capacity
FVC: forced vital capacity
GOLD: Global Initiative for Chronic Obstructive Lung Disease
IND/GLY: indacaterol/glycopyrronium
IQR: interquartile range
LABA: long‐acting beta agonist
LADB: long‐acting dual bronchodilation
LAMA: long‐acting muscarinic antagonist
LLN: lower limit of normal
LMM: linear mixed model
LS: least squares
OR: odds ratio
PEF: peak expiratory flow
RV: residual volume
RV/TLC: residual volume to total lung capacity ratio
TLC: total lung capacity
UMEC/VI: umeclidinium/vilanterol

## 1 Introduction

Chronic obstructive pulmonary disease (COPD) is a major global respiratory disorder, contributing substantially to morbidity and mortality worldwide [1, 2]. In China, COPD affects over 45 million individuals, with an age‐standardized prevalence of 24.0% [3, 4, 5]. International and national guidelines, including those published by the Global Initiative for Chronic Obstructive Lung Disease (GOLD), recommend the combination of long‐acting muscarinic antagonists (LAMAs) and long‐acting β2‐agonists (LABAs) as initial treatment for patients in Group B (0–1 moderate exacerbations and moderate or severe dyspnea) and Group E (≥ 2 moderate or ≥ 1 severe exacerbations, regardless of the degree of dyspnea) [6]. Single‐inhaler long‐acting dual bronchodilators (LADBs) combining LABA and LAMA are widely adopted due to their convenience and improved adherence [7]. While clinical trials [8, 9, 10, 11] have established LADBs' efficacy in symptom relief, exacerbation reduction, and lung function improvement, real‐world effectiveness in Chinese patients remains understudied.

Unlike controlled trials, real‐world clinical practice introduces variables such as adherence, inhaler technique, and patient awareness, which can substantially influence treatment outcomes [12]. Additionally, existing observational studies are limited by short follow‐up periods (24 weeks to 1 year), failing to assess long‐term benefits or disease progression [13, 14, 15]. Most evidence relies on FEV1 as the primary endpoint, yet this metric alone does not fully capture COPD's spirometric functional impairment or pathophysiological complexity [16]. While parameters like forced vital capacity (FVC), inspiratory capacity, and residual volume (RV) are occasionally included, broader spirometry evaluations remain underutilized [13]. Furthermore, while COPD has traditionally been considered a disease of irreversible airflow limitation, the potential for FEV1/FVC reversal after bronchodilator therapy remains poorly understood.

This study evaluated the real‐world effectiveness of single‐inhaler LABA/LAMA therapy in Chinese patients with COPD with a specific focus on spirometry outcomes. Our objectives were threefold: (1) to characterize the longitudinal effects of this therapy on a comprehensive set of pulmonary function parameters, (2) to examine potential associations between baseline characteristics and spirometric treatment outcomes, and (3) to investigate the previously unexplored phenomenon of changes in the FEV1/FVC ratio following dual bronchodilator therapy. The results yield clinically relevant evidence regarding the durability of LADB benefits on lung function and contribute data toward understanding its impact on spirometric disease trajectory.

## 2 Material and Methods

This was a single‐country (China), single‐center, retrospective, noninterventional, real‐world study to evaluate the effectiveness of single‐inhaler LADB (umeclidinium/vilanterol [UMEC/VI] 62.5/25 μg or indacaterol/glycopyrronium [IND/GLY] 110/50 μg, administered once daily) in improving clinical outcomes in patients with COPD compared with baseline.

**FIGURE 1:**
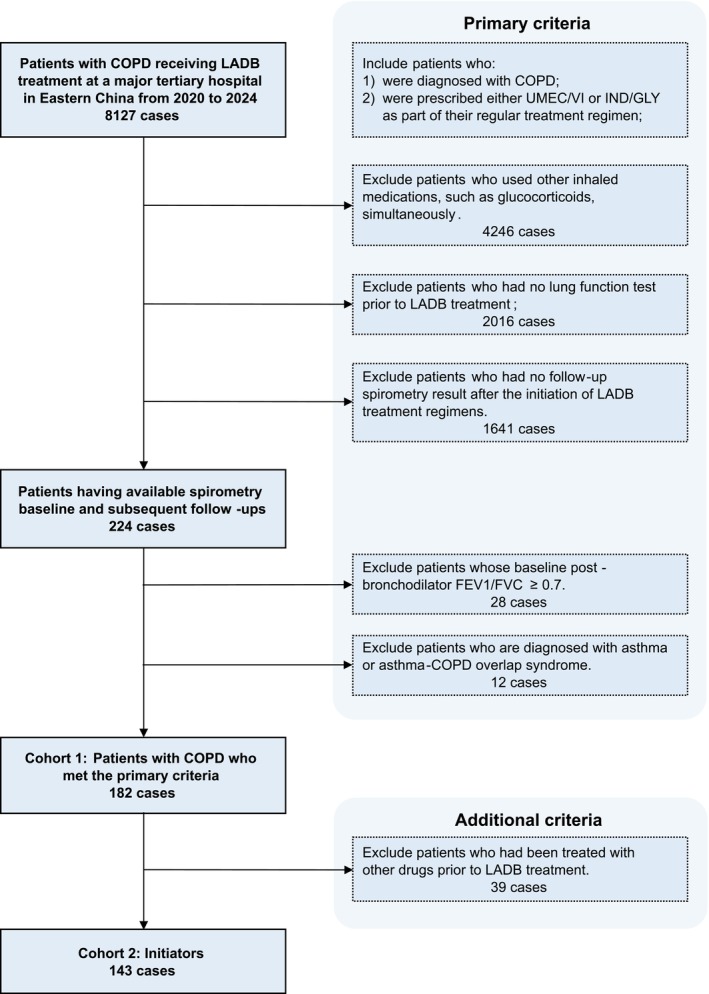
Study design flowchart. COPD: chronic obstructive pulmonary disease; IND/GLY: indacaterol/glycopyrronium; LADB: long‐acting dual bronchodilators; UMEC/VI: umeclidinium/vilanterol.

Eligible patients were identified from the electronic medical records system at a major tertiary hospital in Eastern China from 2020 to 2024 according to the inclusion criteria shown in Figure 1. To align with the study objective of evaluating LABA/LAMA dual therapy in the appropriate clinical population, we excluded patients who were concurrently prescribed inhaled corticosteroid (ICS)–containing regimens. As asthma features reversible obstruction in spirometry, we excluded patients with asthma or asthma‐COPD overlap syndrome to avoid false‐positive lung function improvement. In addition to the primary included population, an initiator subgroup was identified as patients newly diagnosed with COPD who had no prior use of any inhalers. This subgroup was analyzed separately to assess how newly diagnosed, treatment‐naive patients responded to the LADBs. All analyses employed the fixed‐ratio criterion (postbronchodilator FEV1/FVC < 70%) for COPD diagnosis and efficacy assessment per GOLD guidelines [6], unless explicitly stated otherwise for analyses using lower limit of normal (LLN) criteria. Throughout this study, “Sex” refers specifically to birth‐assigned sex (male/female) as recorded in medical records.

The primary outcome was changes in trough FEV1 and FEV1/FVC from baseline at multiple time points. Secondary outcomes were changes in other spirometry parameters from baseline. These analyses on spirometry results were performed using a linear mixed model (LMM) including covariates of baseline spirometry value, age, sex, treatment, follow‐up time (modeled using splines), follow‐up time by treatment, GOLD grades, smoking status, and bronchodilator response (BDR). Least squares (LS) mean change from baseline and 95% confidence intervals (CIs) were presented. There analyses were conducted for both overall population and the initiator subgroup. Subgroup analyses were performed to evaluate differences in spirometry improvements based on baseline characteristics, including age, sex, treatment, GOLD grades, smoking status, and BDR. Pairwise comparisons within each subgroup based on the LMM approach were conducted to control for potential confounders.

A post hoc analysis assessed FEV1/FVC reversal as a binary outcome, defined as an improvement to either: (1) > 70% or (2) above the LLN based on GLI‐2012 equations [17], implemented via the rspiro R package [18]. The analysis of the reversal of FEV1/FVC used either Student's *t* test or chi‐square tests, as appropriate, to compare differences between groups. Univariable logistic regression models were fitted separately for each prespecified baseline variable to assess the likelihood of achieving the reversal of FEV1/FVC based on different variables separately, with odds ratios (ORs) and 95% CI presented. This analysis was considered exploratory due to the limited number of outcome events.

All statistical analyses were performed using R version 4.4.2 (The R Project for Statistical Computing, www.r‐project.org), with statistical significance defined as two‐sided *p* value < 0.05.

This study was conducted in accordance with the amended Declaration of Helsinki. Ethics Committee of Zhongshan Hospital Fudan University approved the protocol (B2025‐170), and written informed consent was obtained from all patients.

## 3 Results

### 3.1 Baseline Characteristics and Follow‐Up

A total of 182 patients with COPD meeting the inclusion and exclusion criteria were identified and included in the analyses (Figure 1). The median follow‐up duration was 257 days (range: 7 days to 4 years; interquartile range [IQR]: 122.75–621.5 days). All 182 patients had at least one follow‐up; 46 of them had two follow‐ups, and 12 of them had three follow‐ups or more. Among the cohort, 143 (78.6%) patients were initiators of single‐inhaler long‐acting dual bronchodilators. Baseline characteristics of the total population and initiators were summarized in Table 1. Baseline pulmonary test results were listed in Table S1.

**TABLE 1:** Baseline patient demographics and characteristics.

Demographics and characteristics		Overall (*N* = 182)	Initiators (*N* = 143)
Sex	Male, *N* (%) female, *N* (%)	150 (82.4) 32 (17.6)	120 (83.9) 23 (16.1)
Age (year), mean ± SD	—	69.4 ± 8.8	69.2 ± 9.3
Smoking status	Current, *N* (%) Former, *N* (%) Never, *N* (%) Unknown, *N* (%)	56 (30.8) 49 (26.9) 37 (20.3) 40 (22.0)	52 (36.4) 36 (25.2) 25 (17.5) 30 (21.0)
Comorbidities	Any of predetermined, *N* (%)	130 (71.4)	106 (74.1)
Bronchiectasis	With, *N* (%) Without, *N* (%)	20 (11.0) 162 (89.0)	15 (10.5) 128 (89.5)
Treatment	IND/GLY, *N* (%) UMEC/VI, *N* (%)	122 (67.0) 60 (33.0)	101 (70.6) 42 (29.4)
Baseline treatment	None, *N* (%) Any prior treatment, *N* (%)	143 (78.6) 39 (21.4)	143 (100) —
GOLD grade	1, *N* (%) 2, *N* (%) 3, *N* (%) 4, *N* (%)	10 (5.5) 104 (57.1) 60 (33.0) 8 (4.4)	9 (6.3) 82 (57.3) 46 (32.2) 6 (4.2)
BDR	Negative, *N* (%) Positive, *N* (%) NA, *N* (%)	108 (59.3) 12 (6.6) 62 (34.1)	92 (64.3) 11 (7.7) 40 (28.0)

Abbreviations: BDR: bronchodilator response; NA: not applicable; SD: standard deviation.

### 3.2 Improvement in FEV1 and FEV1/FVC

**FIGURE 2:**
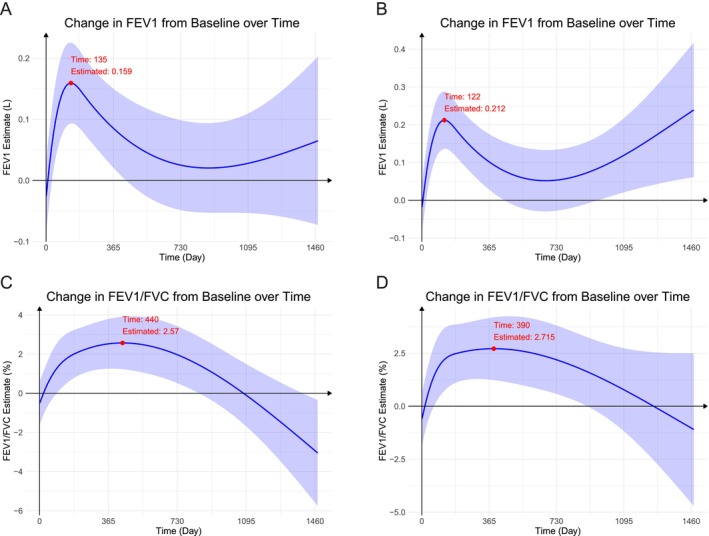
Changes in lung function parameters after treatment. (A) Estimated change in trough forced expiratory volume in 1 s (FEV1) from baseline over time in the entire cohort (*N* = 182). (B) Change in trough FEV1 in initiators (patients receiving first‐time therapy, *N* = 143). (C) Change in FEV1/forced vital capacity (FVC) ratio in the entire cohort. (D) Change in FEV1/FVC ratio in initiators. Solid lines represent estimated changes from linear mixed‐effects models; shaded areas indicate 95% confidence intervals. Baseline values were adjusted for baseline spirometry value, age, sex, treatment, follow‐up time (modeled using splines), follow‐up time by treatment, GOLD grades, smoking status and bronchodilator response.

In the entire patient cohort, the LS mean change in trough FEV1 post‐treatment was 0.154 L [95% CI: 0.090, 0.218] at 24 weeks. Further improvements were observed at 1 year (0.085 L [95% CI: 0.021, 0.150]), 2 years (0.025 L [95% CI: −0.048, 0.099]) and 3 years (0.028 L [95% CI: −0.053, 0.109]). The posttreatment FEV1 initially increased, peaking at 0.159 L at 135 days, followed by a slight decline and stabilization (Figure 2A). Among initiators, the improvement in FEV1 was 0.197 L [95% CI: 0.125, 0.269] at 24 weeks, 0.102 L [95% CI: 0.028, 0.175] at 1 year, 0.053 L [95% CI: −0.028, 0.134] at 2 years, and 0.119 L [95% CI: 0.021, 0.217] at 3 years. A similar pattern of FEV1 changes over time was depicted in Figure 2B, with a peak of 0.212 L at 122 days. Additionally, the duration of FEV1 improvement above the minimally clinically important difference (≥ 100 mL) ranged from approximately 67–215 days.

In the entire patient cohort, the LS mean change in trough FEV1/FVC post‐treatment was 1.93 [95% CI: 0.694, 3.16] at 24 weeks, 2.52 [95% CI: 1.26, 3.79] at 1 year, 1.97 [95% CI: 0.521, 3.42] at 2 years, and −0.0983 [95% CI: −1.69, 1.49] at 3 years. The posttreatment FEV1/FVC reached its peak at 440 days, later than the peak observed for FEV1. It remained at a relatively stable and high level until approximately 2 years, after which it gradually declined (Figure 2C). Among initiators, FEV1/FVC improvement was 2.44 [95% CI: 1.04, 3.84] at 24 weeks, 2.71 [95% CI: 1.26, 4.16] at 1 year, 2.19 [95% CI: 0.58, 3.8] at 2 years and 0.778 [95% CI: −1.18, 2.73] at 3 years. The pattern of the change of FEV1/FVC in initiators (Figure 2D) was similar to that in the entire patient cohort.

**FIGURE 3:**
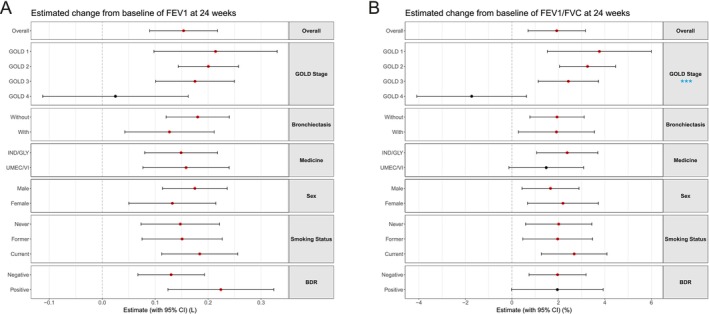
Subgroup analyses of lung function changes at 24 weeks. (A) Change in trough forced expiratory volume in 1 s (FEV1) from baseline. (B) Change in FEV1/forced vital capacity (FVC) ratio from baseline. Red dots indicate statistically significant changes compared with baseline (i.e., 0) (*p* < 0.05), while black dots represent no statistically significant change. Blue stars highlight statistically significant differences within subgroups. Sex categories were limited to binary classification per data source requirements. BDR: bronchodilator response; CI: confidence interval; IND/GLY: indacaterol/glycopyrronium; UMEC/VI: umeclidinium/vilanterol; ***: *p* < 0.001.

Subgroup analysis based on baseline demographics and clinical characteristics demonstrated that FEV1 improvement was observed in most subgroups, except for patients classified as GOLD 4 (Figure 3A). Similarly, improvements in FEV1/FVC were noted across most subgroups (Figure 3B). An earlier stage in GOLD was associated with a greater improvement in trough FEV1/FVC (*p* < 0.001). No significant differences in FEV1 and FEV1/FVC improvement were found among the other subgroups.

### 3.3 Improvement of Other Spirometry Parameters

The entire cohort showed time‐dependent improvements in spirometry parameters (Figure S1). LADB treatment improved ventilation, small airway function, and reduced RV, though diffusion remained unchanged. Significant changes occurred at multiple time points, with 24‐week and 1‐year data detailed in Table 2 (initiator subgroup results in Table S2).

**TABLE 2:** Estimated change of spirometry parameters at 24 weeks and 1 year in the entire cohort.

Parameter	24 weeks		1 year	
	Change [95% CI]	*p* ^†^	Change [95% CI]	*p* ^†^
FVC	0.173 L, [0.089, 0.258]	**< 0.001**	0.020 L, [−0.064, 0.105]	0.635
FVC %pred	6.173%, [3.804, 8.542]	**< 0.001**	2.221%, [−0.177, 4.62]	0.069
FEV1	0.154 L, [0.09, 0.218]	**< 0.001**	0.085 L, [0.021, 0.15]	**0.010**
FEV1 %pred	6.810%, [4.573, 9.047]	**< 0.001**	4.984%, [2.697, 7.272]	**< 0.001**
FEV1/FVC	1.929%, [0.694, 3.164]	**0.002**	2.525%, [1.262, 3.788]	**< 0.001**
FEV1/FVC %pred	2.635%, [0.948, 4.321]	**0.002**	3.688%, [1.963, 5.414]	**< 0.001**
TLC	−0.014 L, [−0.144, 0.116]	0.836	−0.156 L, [−0.289, −0.023]	**0.022**
TLC %pred	0.097%, [−1.916, 2.111]	0.924	−1.276%, [−3.349, 0.797]	0.227
RV	−0.142 L, [−0.254, −0.031]	**0.012**	−0.140 L, [−0.255, −0.025]	**0.017**
RV %pred	−8.713%, [−13.757, −3.67]	**0.001**	−5.529%, [−10.712, −0.346]	**0.037**
RV/TLC	−2.816%, [−4.466, −1.166]	**0.001**	−0.617%, [−2.297, 1.062]	0.470
PEF	0.491 L/s, [0.261, 0.72]	**< 0.001**	0.257 L/s, [0.024, 0.49]	**0.031**
PEF %pred	7.244%, [4.569, 9.919]	**< 0.001**	4.647%, [1.902, 7.392]	**0.001**
FEF25	0.477 L/s, [0.288, 0.665]	**< 0.001**	0.329 L/s, [0.137, 0.522]	**0.001**
FEF25 %pred	7.128%, [4.462, 9.794]	**< 0.001**	5.239%, [2.514, 7.964]	**< 0.001**
FEF50	0.210 L/s, [0.129, 0.292]	**< 0.001**	0.172 L/s, [0.089, 0.255]	**< 0.001**
FEF50 %pred	5.649%, [3.616, 7.682]	**< 0.001**	4.845%, [2.767, 6.923]	**< 0.001**
FEF75	0.062 L/s, [0.032, 0.093]	**< 0.001**	0.047 L/s, [0.017, 0.078]	**0.003**
FEF75 %pred	8.431%, [5.525, 11.337]	**< 0.001**	8.126%, [5.173, 11.079]	**< 0.001**
DLCO/VA	0.038 mmol/min/kPa/L, [−0.015, 0.091]	0.156	0.027 mmol/min/kPa/L, [−0.027, 0.081]	0.334
DLCO/VA %pred	3.228%, [−0.61, 7.065]	0.099	2.033%, [−1.903, 5.969]	0.310

Abbreviations: DLCO/VA: diffusing capacity per unit alveolar volume; FEF: forced expiratory flow; FEV1: forced expiratory volume in 1 s; FEV1/FVC: ratio of forced expiratory volume in 1 s to forced vital capacity; FVC: forced vital capacity; PEF: peak expiratory flow; RV: residual volume; RV/TLC: residual volume to total lung capacity ratio; TLC: total lung capacity; %pred: percent predicted.

^†^ The *p* values were computed using the estimated marginal means obtained from the linear mixed‐effects model, implemented via the emmeans R package. These *p* values reflect the significance of the estimated means relative to a null hypothesis value of 0, with statistical significance defined as *p* < 0.05. Values in bold indicate statistically significant results (*p* < 0.05).

Subgroup analyses, with demographic and clinical characteristics modeled as fixed effects in LMM, revealed both similarities and differences in treatment response. The corresponding *p* values (indicating subgroup differences) are presented in Table S3, while estimated changes for each subgroup are shown in Figure S2. GOLD grade significantly influenced outcomes, with lower grades correlating to better responses in FEV1/FVC, RV/TLC, PEF, and FEF75. Medication type and concomitant bronchiectasis had no significant effects.

### 3.4 Reversal of FEV1/FVC

We identified a population of 21 patients (11.5%) whose posttreatment trough FEV1/FVC ratio improved to over 70%, referred to as the “reversed group.” The median follow‐up time until the reversal of FEV1/FVC was 179 days, with the 25th and 75th percentiles at 85 and 429 days, respectively. Only two patients demonstrated positive BDR. Detailed information is provided in Table S4. We additionally assessed FEV1/FVC reversal using the LLN criteria. Among 154 patients meeting LLN‐based COPD criteria, 20 (13.0%) achieved postbronchodilator FEV1/FVC above their individualized LLN thresholds (Table 3).

**TABLE 3:** Posttreatment FEV1/FVC reversal in patients with COPD by different criteria.

Criterion	Patients with COPD^a^ (*N*)	FEV1/FVC reversed to normal^b^ (*N*, %)	Subcategories of patients whose FEV1/FVC reversed	
			FEV1 remaining impaired^c^ (*N*, %)	FEV1 reserved to normal^d^ (*N*, %)
Fixed‐ratio	182	21, 11.5%	11, 6.0%	10, 5.5%
LLN	154	20, 13.0%	14, 9.1%	6, 3.9%

^a^ Postbronchodilator FEV1/FVC < 0.7 (fixed‐ratio) or lower limit of normal (LLN).

^b^ FEV1/FVC ≥ 0.7 (fixed‐ratio) or LLN.

^c^ Trough FEV1 < 80% predicted (fixed‐ratio) or LLN, meeting criteria for preserved ratio impaired spirometry group.

^d^ Trough FEV1 ≥ 80% predicted (fixed‐ratio) or LLN. Percentages in the table were calculated based on the total number of patients with COPD in each category.

**FIGURE 4:**
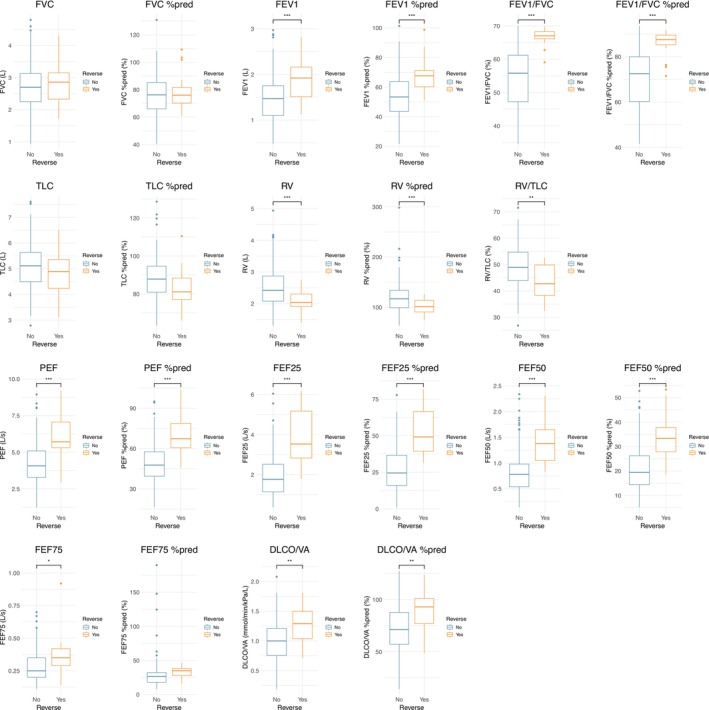
Comparison of baseline lung function between nonreversed (No) and reversed (Yes) groups. Boxes represent interquartile range (IQR) with median line; whiskers extend to 1.5 × IQR; circles indicate outliers. Group difference was statistically significant (*p* < 0.05) by Student's *t* test. DLCO/VA: diffusing capacity per unit alveolar volume; FEF: forced expiratory flow; FEV1: forced expiratory volume in 1 s; FEV1/FVC: ratio of forced expiratory volume in 1 s to forced vital capacity; FVC: forced vital capacity; PEF: peak expiratory flow; RV: residual volume; RV/TLC: residual volume to total lung capacity ratio; TLC: total lung capacity; %pred: percent predicted.

Given the wider clinical adoption of fixed‐ratio criteria, all subsequent analyses focused on FEV1/FVC reversal to above 70%. Baseline demographic and clinical characteristics showed no significant differences (*p* > 0.05) except for GOLD grades (*p* = 0.001), which were less severe in the reversed group. Incidence rate was 30% and 17.3% in patients at GOLD 1 and 2, respectively. Additionally, the reversed group exhibited better baseline lung function than the nonreversed group, including FEV1, FEV1%pred, FEV1/FVC, and FEV1/FVC %pred, RV, RV %pred, RV/TLC, PEF, PEF %pred, FEF25, FEF25%pred, FEF50, FEF50%pred, FEF75, DLCO/VA, and DLCO/VA %pred (Figure 4).

**FIGURE 5:**
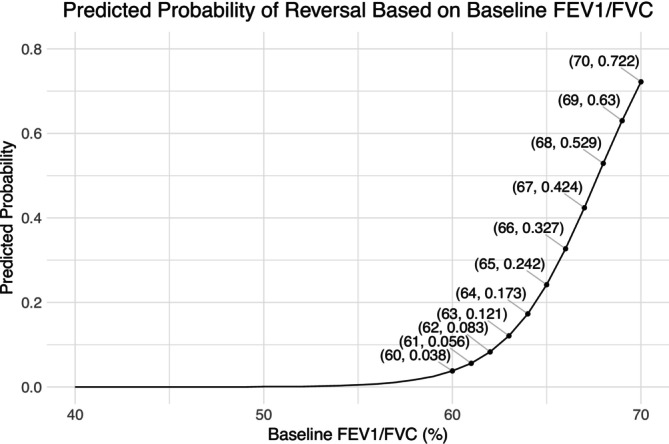
Predicted probability of FEV1/FVC reversal to above 70% based on baseline FEV1/FVC. Points show predicted probabilities at FEV1/FVC ≥ 60% (labeled values); solid line represents logistic regression fit. FEV1/FVC: forced expiratory volume in 1 s to forced vital capacity ratio.

Logistic regression analysis revealed that better ventilation function parameters, lower RV metrics, and better diffusion function were significantly associated with a higher likelihood of the reversal of FEV1/FVC after treatment (Table S5). Among these variables, the baseline FEV1/FVC ratio had the strongest influence, with an OR of 51.68 for each scaled unit increase in baseline FEV1/FVC. Use the not scaled data, the OR for 1 unit of increase in baseline FEV1/FVC was 1.52 [95% CI: 1.29, 1.89]. The probability of the reversal of FEV1/FVC after treatment increased as the baseline FEV1/FVC ratio increased (Figure 5). For instance, an increase in baseline FEV1/FVC from 60 to 65 was associated with a 20.4% increase in the likelihood of reversal. In our study cohort, the incidence rate of FEV1/FVC reversal was 0.9%, 6.3%, and 50% among patients with baseline FEV1/FVC ratios below 60%, between 60% and 65%, and above 65%, respectively.

Using a cutoff value of 60 for FEV1/FVC, we categorized the population into two groups: those with a baseline FEV1/FVC greater than 60 and those with a baseline FEV1/FVC less than or equal to 60. The OR for FEV1/FVC reversal in the group with a baseline FEV1/FVC > 60 was significantly higher compared with the group with FEV1/FVC ≤ 60 (OR = 47.1, 95% CI: 9.4, 856.9). The area under the curve for predicting FEV1/FVC reversal based on this cutoff was 0.8271.

## 4 Discussion

This real‐world study evaluated the effectiveness of single‐inhaler LADB treatment (IND/GLY and UMEC/VI) in improving lung function in patients with COPD. The results demonstrated that LADB significantly improved ventilation, enhanced small airway function, and reduced RV. Specifically, posttreatment FEV1 improved by 0.154 L at 24 weeks, 0.085 L at 1 year, 0.025 L at 2 years, and 0.028 L at 3 years in the entire patient cohort. The improvement in FEV1 at 24 weeks was consistent with findings from previous studies, such as 0.122 L in the EMAX trial with UMEC/VI [14] and 0.194 L with IND/GLY [15]. Given that FEV1/FVC has been underreported in previous studies, this study demonstrated a significant increase in FEV1/FVC (1.93 at 24 weeks, 2.52 at 1 year, and 1.97 at 2 years), thereby contributing novel insights into improvements in this critical spirometry parameter.

One previous real‐world observational study of LADBs reported that both UMEC/VI and IND/GLY improved FEV1, with UMEC/VI also improving FVC, but neither showed improvement in RV [13]. However, in this current study, FEV1, FVC, and RV all improved, regardless of the medication used. Additionally, no significant differences were found in the ability of either medication to improve lung function. These discrepancies in results may be explained by differences in study designs, patient cohorts, and statistical methods.

In addition to ventilation parameters, this study also focused on small airway function and hyperinflation, key pathological features of COPD that have often been underexplored in prior research [19]. Improvements in FEF25, FEF50, and FEF75 suggested that LADB treatment was effective in enhancing small airway function. The reduction in RV and RV/TLC further indicated that LADB therapy contributed to a decrease in hyperinflation, an important aspect of COPD management.

The study also identified a cohort of initiators, patients who had not received prior treatment before starting LADB therapy. This group showed greater improvement in lung function, with a mean FEV1 increase of 0.197 L at 24 weeks, compared with 0.154 L for the entire cohort. However, statistical comparisons were not performed between these populations due to overlap.

Subgroup analyses based on demographic and clinical characteristics revealed that a lower GOLD grade was associated with better treatment responses across various parameters, such as FEV1/FVC, RV/TLC, PEF, and FEF75. Other subgrouping factors exhibited limited or negligible effects on treatment outcomes. Although bronchiectasis is a common comorbidity in COPD linked to accelerated lung function decline and increased mortality [20, 21], our study did not detect significant differences in LADB response in this subgroup. Previous research by Vogelmeier et al. [22] suggested that patients with COPD with greater bronchodilator reversibility typically show better treatment responses. While our data showed a similar trend in FEV1 improvement, the difference did not reach statistical significance, possibly due to the limited sample size.

One unique aspect of this study was its longitudinal analysis of post‐treatment lung function over a period of up to 4 years. The data revealed that posttreatment FEV1 initially increased, peaking at 0.159 L at 135 days, followed by a slight decline and eventual stabilization over a longer period, up to approximately 4 years. Other parameters analyzed followed a similar trend, with peaks around the 6‐month mark, while FEV1/FVC showed a slower improvement, peaking at 440 days and remaining at a relatively stable and high level until approximately 2 years. In the natural history, FEV1 falls gradually over a lifetime and COPD generally accelerates the decline [23]. However, our study identified a sustained stabilization of FEV1 over an extended observation period. While these findings should be interpreted with caution due to the limited sample size and consequent wide CIs, they suggested that prolonged use of LADBs may provide enduring benefits by attenuating or potentially reversing the characteristic decline in lung function among patients with COPD.

COPD is a disease characterized by incompletely reversible expiratory airflow limitation that is indexed by postbronchodilator FEV1/FVC < 70% according to the GOLD diagnosis criteria [6]. It was believed that FEV1/FVC cannot be incompletely reversed. Here, this study reported a group of 21 patients (11.5%) whose FEV1/FVC were improved to over 70% after LADB treatment for the first time. This study also vation period. While these findings should be interpreted with caution due to the limited sample size and consequent wide CIs, they suggested that prolonged use of LADBs may provide enduring benefits by attenuating or potentially reversing the characteristic decline in lung function among patients with COPD.

COPD is a disease characterized by incompletely reversible expiratory airflow limitation that is indexed by postbronchodilator FEV1/FVC < 70% according to the GOLD diagnosis criteria [6]. It was believed that FEV1/FVC cannot be incompletely reversed. Here, this study reported a group of 21 patients (11.5%) whose FEV1/FVC were improved to over 70% after LADB treatment for the first time. This study also used the LLN diagnosis criteria recommended by ATS/ERS [24] for further confirmation, according to which 13.0% patients' posttreatment FEV1/FVC surpassed the LLN. The concordance between both diagnostic standards (fixed‐ratio and LLN) provides evidence for the existence of FEV1/FVC reversal after treatment in this subset of patients. Moreover, this study revealed that patients with better baseline lung function were more likely to achieve this reversal. And exploratory logistic regression identified baseline FEV1/FVC as the strongest predictor for this change, with an OR of 1.52 [95% CI: 1.29, 1.89] per unit (%) increase. Patients with a baseline FEV1/FVC > 60% had a markedly higher likelihood (OR = 47.1, 95% CI: 9.4, 856.9) of achieving a posttreatment ratio above 70% compared with those with a baseline ratio ≤ 60%. Nonetheless, these findings must be interpreted with caution for several reasons. First, the observed increase above the fixed threshold may reflect, in part, a combination of measurement variability [25] and the use of a conventional diagnostic cut‐off, rather than a definitive reversal of the underlying pathophysiology. Second, the regression analysis was constrained by a limited number of outcome events (*n* = 21), resulting in imprecise estimates with wide CIs that indicate potential model instability. Despite these weaknesses, the phenomenon raises pertinent clinical questions. For instance, it prompts consideration of whether bronchodilation treatment strategies should be re‐evaluated for patients who no longer meet the spirometric diagnostic criteria for airflow obstruction after therapy. Thus, further studies with larger population are needed to investigate this phenomenon thoroughly.

The promising yet preliminary findings regarding lung function improvement and FEV1/FVC changes of LADB therapy must be considered within the context of several important study limitations. First, as the study was designed to assess LADB therapy, we exclusively enrolled patients on LABA/LAMA regimen, systematically excluding those receiving ICS. Consequently, our cohort likely represented a more stable COPD population, and the results may not extend to higher‐risk patients requiring ICS‐containing therapies, which constituted a major limitation to external validity. Second, the exclusive focus on spirometric outcomes of this study constrained its clinical interpretability. Due to data constraints, this study could not evaluate critical patient‐centered endpoints such as exacerbation rates, symptom burden (e.g., CAT or mMRC scores), or health‐related quality of life, therefore failing to assess other crucial clinical endpoints in COPD management. Further studies including these variables are needed to provide a more comprehensive assessment of treatment efficacy.

There are also other limitations in addition to those discussed before. First, it was a retrospective observational study conducted at a single medical center with a relatively small patient sample, introducing some degree of selection bias. The choice of medication was made by physicians based on clinical judgment, patient preferences, and medication availability, which may have introduced variability. Second, the small number of patients with long‐term follow‐up data contributed to larger error margins in estimating lung function improvements at later time points. Third, the study did not differentiate between COPD phenotypes (emphysema vs. chronic bronchitis), which could influence treatment response. Forth, this study failed to collect data on smoking status changes during the follow‐up period due to inaccessibility, as smoking cessation is also a key modifier of lung function trajectory.

Despite all these limitations, this study provided valuable real‐world evidence on the efficacy of LADB therapy on spirometry outcomes, offering insights into improvements across multiple spirometry parameters and a longitudinal perspective on spirometry outcomes spanning up to 4 years. It suggested the sustained benefits of LADB therapy in enhancing lung function among patients with COPD, including the observation that some patients' FEV1/FVC ratio improved to above 70% post‐treatment. These findings were in line with the emphasis of long‐term treatment strategies in COPD management.

Overall, single‐inhaler LADB therapy, including IND/GLY and UMEC/VI, effectively improved lung function in patients with COPD, with significant improvements in key spirometry parameters such as FEV1, FEV1/FVC, FVC, RV, and indices of small airway function, which were sustained over several years. Patients with lower GOLD grades derived greater improvements in these spirometry measures. Notably, this study reported the reversal of FEV1/FVC to over 70% following LADB treatment observed in a subset of patients, suggesting a potentially meaningful phenomenon in spirometry improvement post treatment. These findings contributed to the understanding of lung function response to dual bronchodilation in a real‐world setting and emphasized the importance of regular long‐term LADB treatment.

## Author Contributions

Y.L., Y.Z., L.X., L.W., C.C., and Y.S. conceived and designed the study. Y.L., Y.Z., L.X., H.T., J.H., Y.Y., Y.G., and W.J. collected the data. Y.L. analyzed the data. Y.L., L.W., C.C., and Y.S. wrote and revised the manuscript. All authors read and approved the final manuscript.

## Funding

This study was supported by Shanghai Three‐year Action Plan to Strengthen the Construction of Public Health System (GWVI‐11.1‐18), National Natural Science Foundation of China (82130001), National Key Research and Development Program of China (2024YFC3044400), R&D Program of Guangzhou National Laboratory (GZNL2024A02003), The Construction of Multi‐Disciplinary Treatment System for Severe Pneumonia (W2020‐013), Shanghai Municipal Science and Technology Major Project (ZD2021CY001), and Shanghai Municipal Key Clinical Specialty (shslczdzk02201).

## Ethics Statement

This study was approved by the Ethics Committee of Zhongshan Hospital Fudan University (B2025‐170). Written informed consent was obtained from patients who could be contacted, and the requirement for informed consent was waived for those who could not be reached, as approved by the committee.

## Conflicts of Interest

The authors declare no conflicts of interest.

## Supporting information


**Data S1:** Supplementary Information.

**Figure S1:** Estimated change in multiple spirometry metrics from baseline over time after treatment in the entire patient cohort. *Solid lines represent estimated changes from linear mixed‐effects models; shaded areas indicate 95% confidence intervals. Baseline values were adjusted for baseline spirometry value, age, sex, treatment, follow‐up time (modeled using splines), follow‐up time by treatment, GOLD grades, smoking status and bronchodilator response.*DLCO/VA: Diffusing Capacity per Unit Alveolar Volume; FEF: Forced Expiratory Flow; FEV1: Forced Expiratory Volume in 1 second; FEV1/FVC: Ratio of Forced Expiratory Volume in 1 s to Forced Vital Capacity; FVC: Forced Vital Capacity; PEF: Peak Expiratory Flow; RV: Residual Volume; RV/TLC: Residual Volume to Total Lung Capacity Ratio; TLC: Total Lung Capacity; %pred: percent predicted.

**Figure S2:** Estimated change in multiple spirometry metrics from baseline at 24 weeks in different subgroups of the entire patient cohort. Red dots indicate statistically significant changes compared to baseline (i.e. 0) (P<0.05), while black dots represent no statistically significant change. Blue stars highlight statistically significant differences within subgroups. IND/GLY: indacaterol/glycopyrronium; UMEC/VI: umeclidinium/vilanterol; BDR: bronchodilator response; CI: confidence interval; *: p < 0.05; **: p < 0.01; ***: p < 0.001.

**Table S1:** Baseline pulmonary function test result.

**Table S2:** Estimated change of spirometry parameters at 24 weeks and 1 year in initiators.

**Table S3:** *P*‐value of fixed effects on spirometry parameters.

**Table S4:** Baseline patient demographics and characteristics of reversed and non‐reversed group.

**Table S5:** Odds ratios for various baseline lung function parameters, demographics, and characteristics in relation to the likelihood of FEV1/FVC reversal.

## Data Availability

The datasets used and analyzed during the current study are available from the corresponding author on reasonable request.
