# Supplementary material for: Temporal and Partial Reversal of Airflow Limitation in Patients With COPD Treated With Single‐Inhaler Long‐Acting Dual Bronchodilators
Source: Clin Respir J. 2026 Apr 20;20(4):e70173. doi: 10.1111/crj.70173 (PMC13096688; doi:10.1111/crj.70173)
Supplement: Supplementary file 1 — Data S1: Supplementary Information. [file CRJ-20-e70173-s005.docx]

**Supplementary Figure 1.** Estimated change in multiple spirometry metrics from baseline over time after treatment in the entire patient cohort.

Solid lines represent estimated changes from linear mixed-effects models; shaded areas indicate 95% confidence intervals. Baseline values were adjusted for baseline spirometry value, age, sex, treatment, follow-up time (modeled using splines), follow-up time by treatment, GOLD grades, smoking status and bronchodilator response.

DLCO/VA: Diffusing Capacity per Unit Alveolar Volume; FEF: Forced Expiratory Flow; FEV1: Forced Expiratory Volume in 1 second; FEV1/FVC: Ratio of Forced Expiratory Volume in 1s to Forced Vital Capacity; FVC: Forced Vital Capacity; PEF: Peak Expiratory Flow; RV: Residual Volume; RV/TLC: Residual Volume to Total Lung Capacity Ratio; TLC: Total Lung Capacity; %pred: percent predicted.

**Supplementary Figure 2.** Estimated change in multiple spirometry metrics from baseline at 24 weeks in different subgroups of the entire patient cohort.

Red dots indicate statistically significant changes compared to baseline (i.e. 0) (P<0.05), while black dots represent no statistically significant change. Blue stars highlight statistically significant differences within subgroups.

IND/GLY: indacaterol/glycopyrronium; UMEC/VI: umeclidinium/vilanterol; BDR: bronchodilator response; CI: confidence interval; *: p<0.05; **: p<0.01; ***: p<0.001.
