# Supplementary figures and images for: Temporal and Partial Reversal of Airflow Limitation in Patients With COPD Treated With Single‐Inhaler Long‐Acting Dual Bronchodilators
Source: Clin Respir J. 2026 Apr 20;20(4):e70173. doi: 10.1111/crj.70173 (PMC13096688; doi:10.1111/crj.70173)

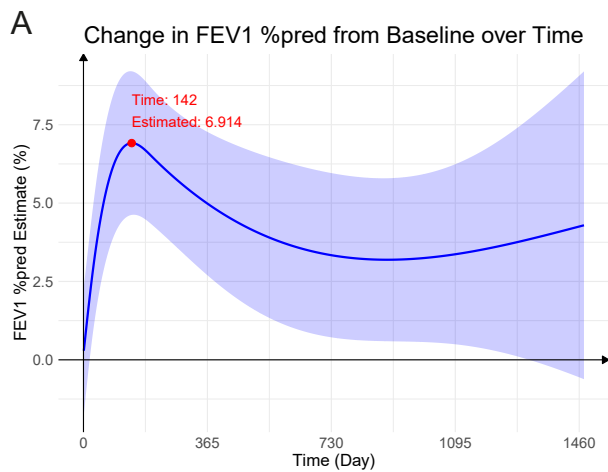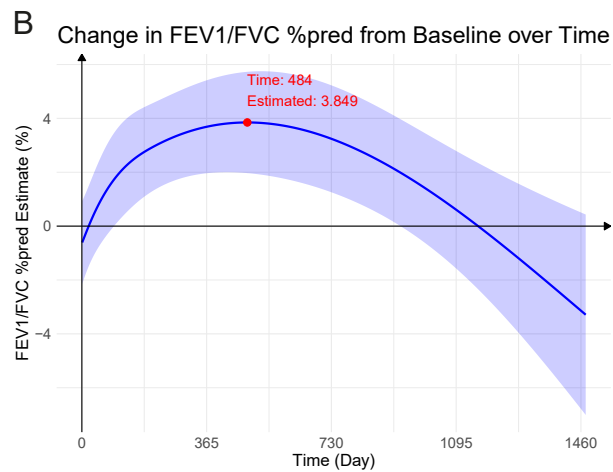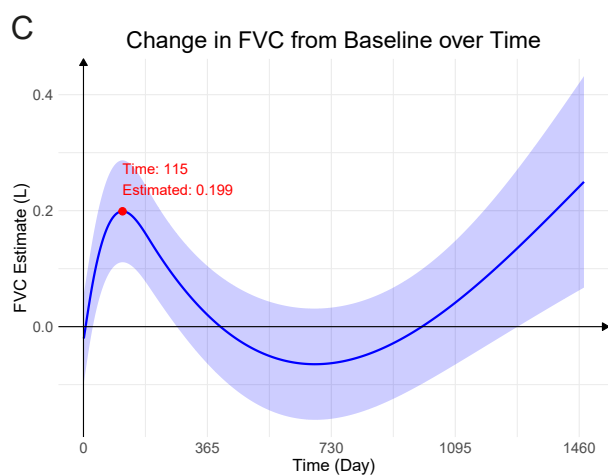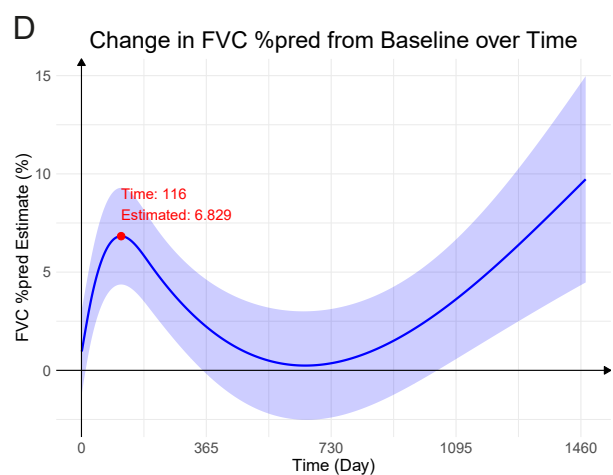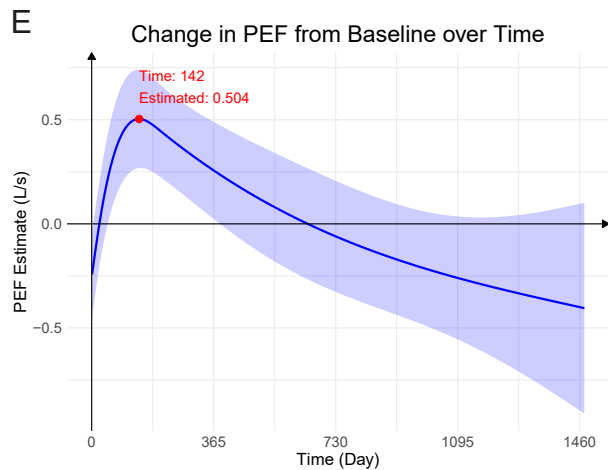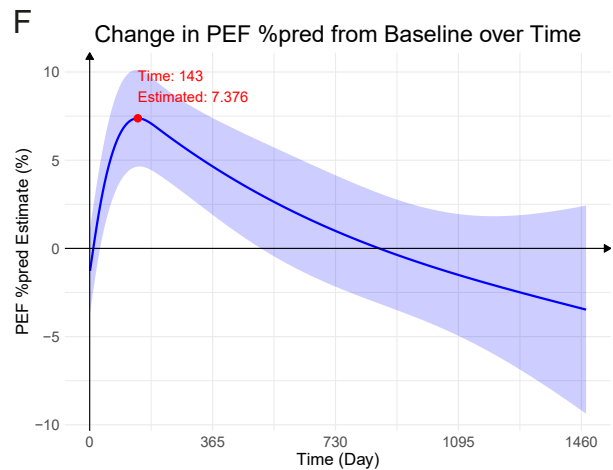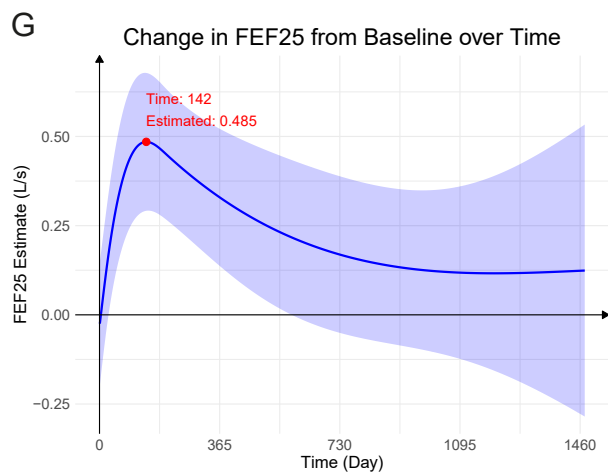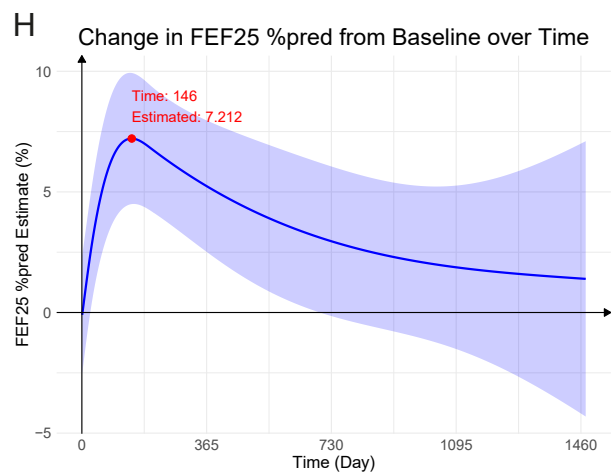

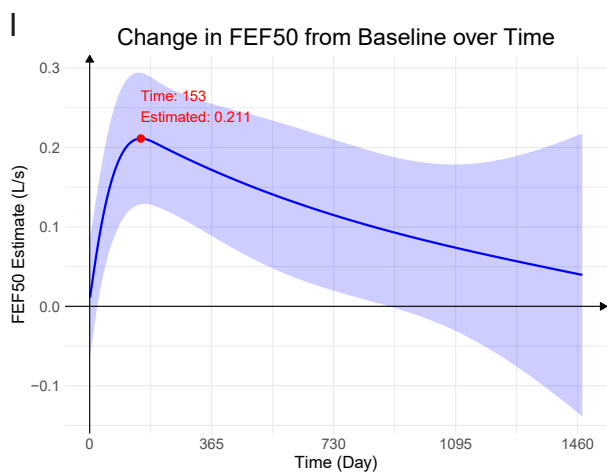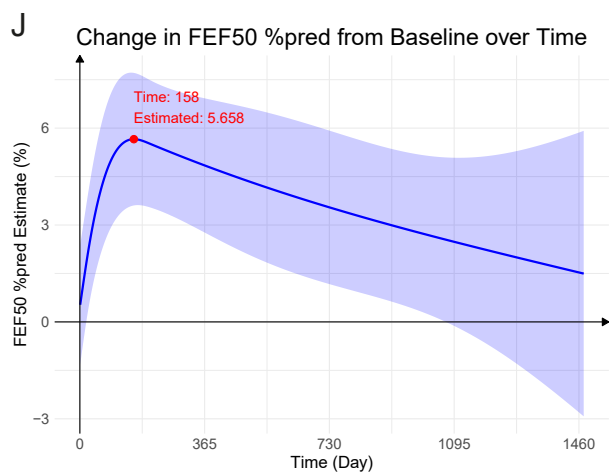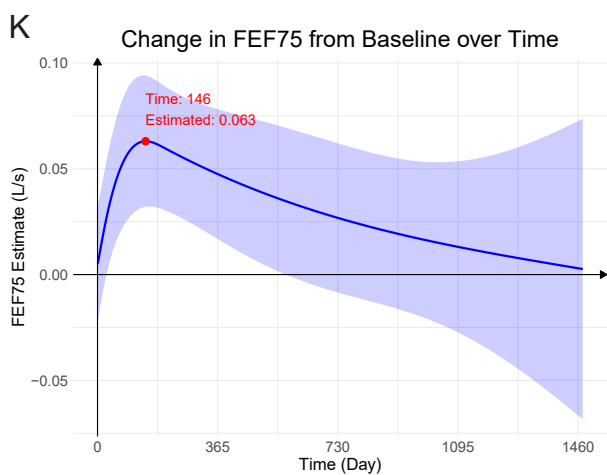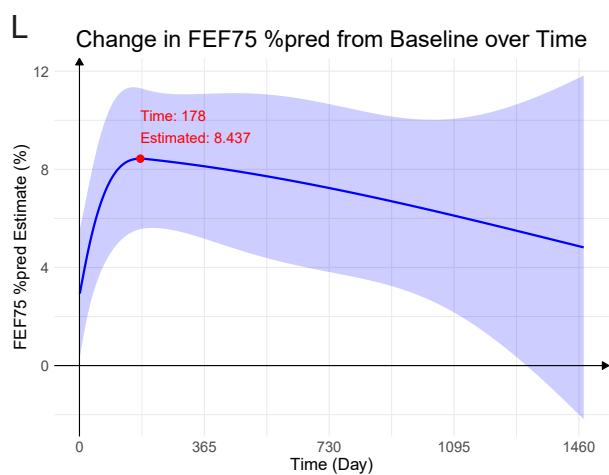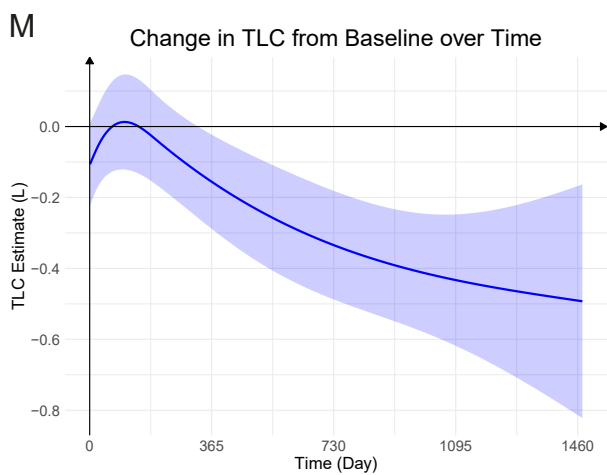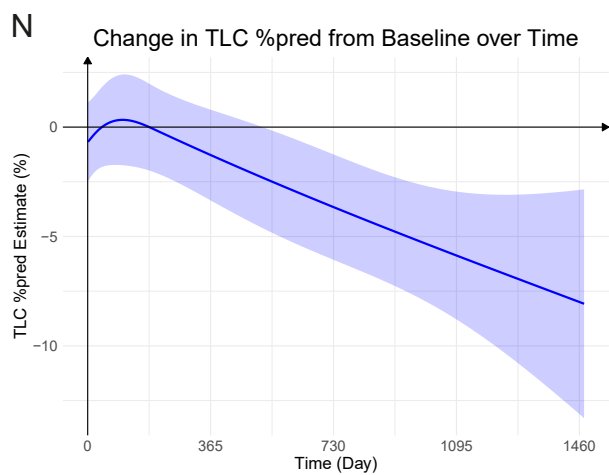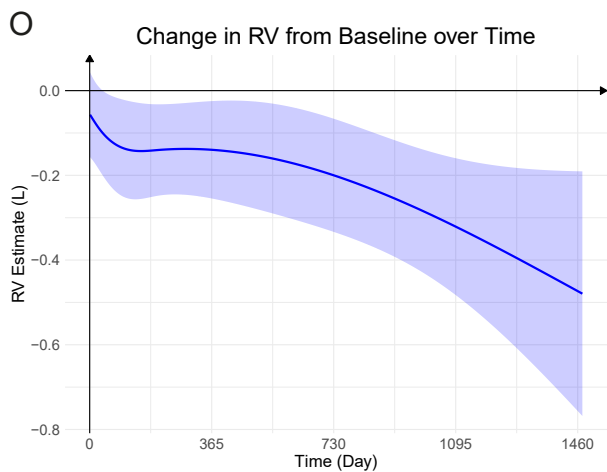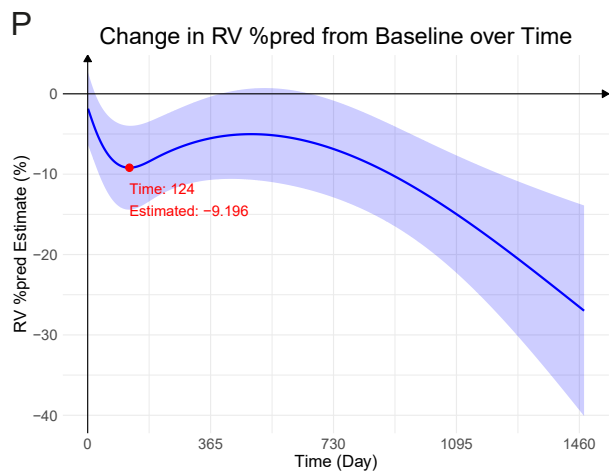

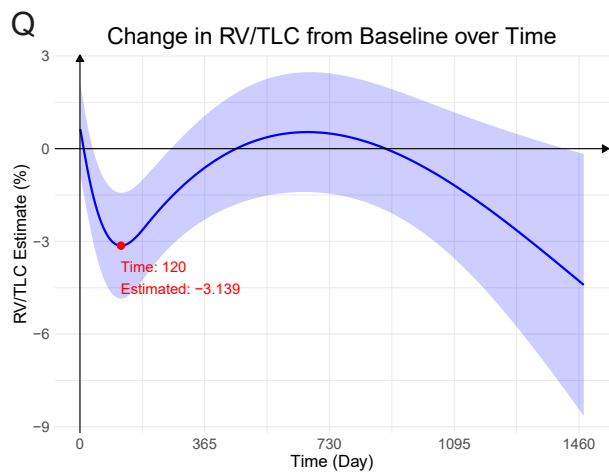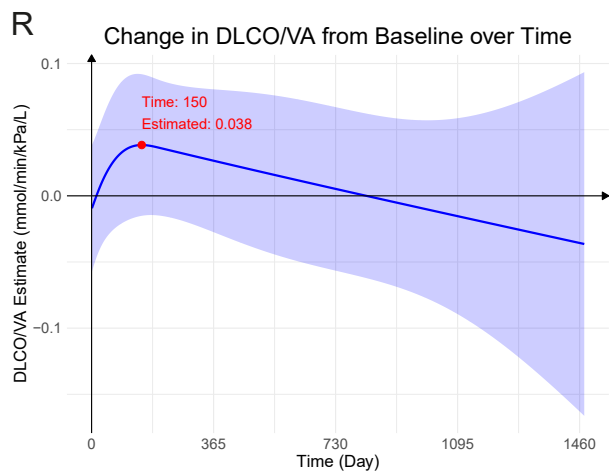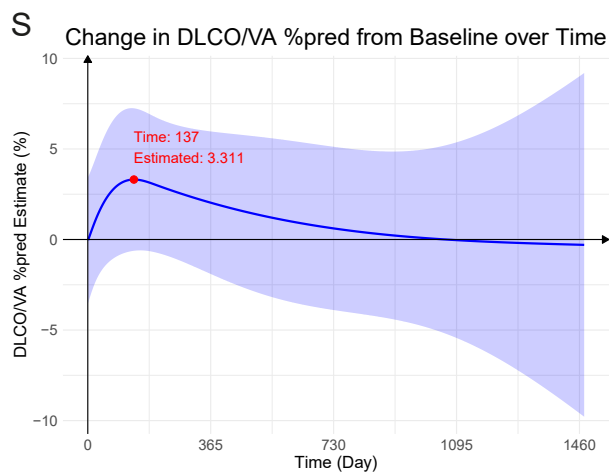

Supplement: Supplementary file 2 — Figure S1: Estimated change in multiple spirometry metrics from baseline over time after treatment in the entire patient cohort. Solid lines represent estimated changes from linear mixed‐effects models; shaded areas indicate 95% confidence intervals. Baseline values were adjusted for baseline spirometry value, age, sex, treatment, follow‐up time (modeled using splines), follow‐up time by treatment, GOLD grades, smoking status and bronchodilator response.DLCO/VA: Diffusing Capacity per Unit Alveolar Volume; FEF: Forced Expiratory Flow; FEV1: Forced Expiratory Volume in 1 second; FEV1/FVC: Ratio of Forced Expiratory Volume in 1 s to Forced Vital Capacity; FVC: Forced Vital Capacity; PEF: Peak Expiratory Flow; RV: Residual Volume; RV/TLC: Residual Volume to Total Lung Capacity Ratio; TLC: Total Lung Capacity; %pred: percent predicted. [file CRJ-20-e70173-s004.pdf]
