# Supplementary material for: Temporal and Partial Reversal of Airflow Limitation in Patients With COPD Treated With Single‐Inhaler Long‐Acting Dual Bronchodilators
Source: Clin Respir J. 2026 Apr 20;20(4):e70173. doi: 10.1111/crj.70173 (PMC13096688; doi:10.1111/crj.70173)
Supplement: Supplementary file 3 — Figure S2: Estimated change in multiple spirometry metrics from baseline at 24 weeks in different subgroups of the entire patient cohort. Red dots indicate statistically significant changes compared to baseline (i.e. 0) (P<0.05), while black dots represent no statistically significant change. Blue stars highlight statistically significant differences within subgroups. IND/GLY: indacaterol/glycopyrronium; UMEC/VI: umeclidinium/vilanterol; BDR: bronchodilator response; CI: confidence interval; *: p < 0.05; **: p < 0.01; ***: p < 0.001. [file CRJ-20-e70173-s001.pdf]

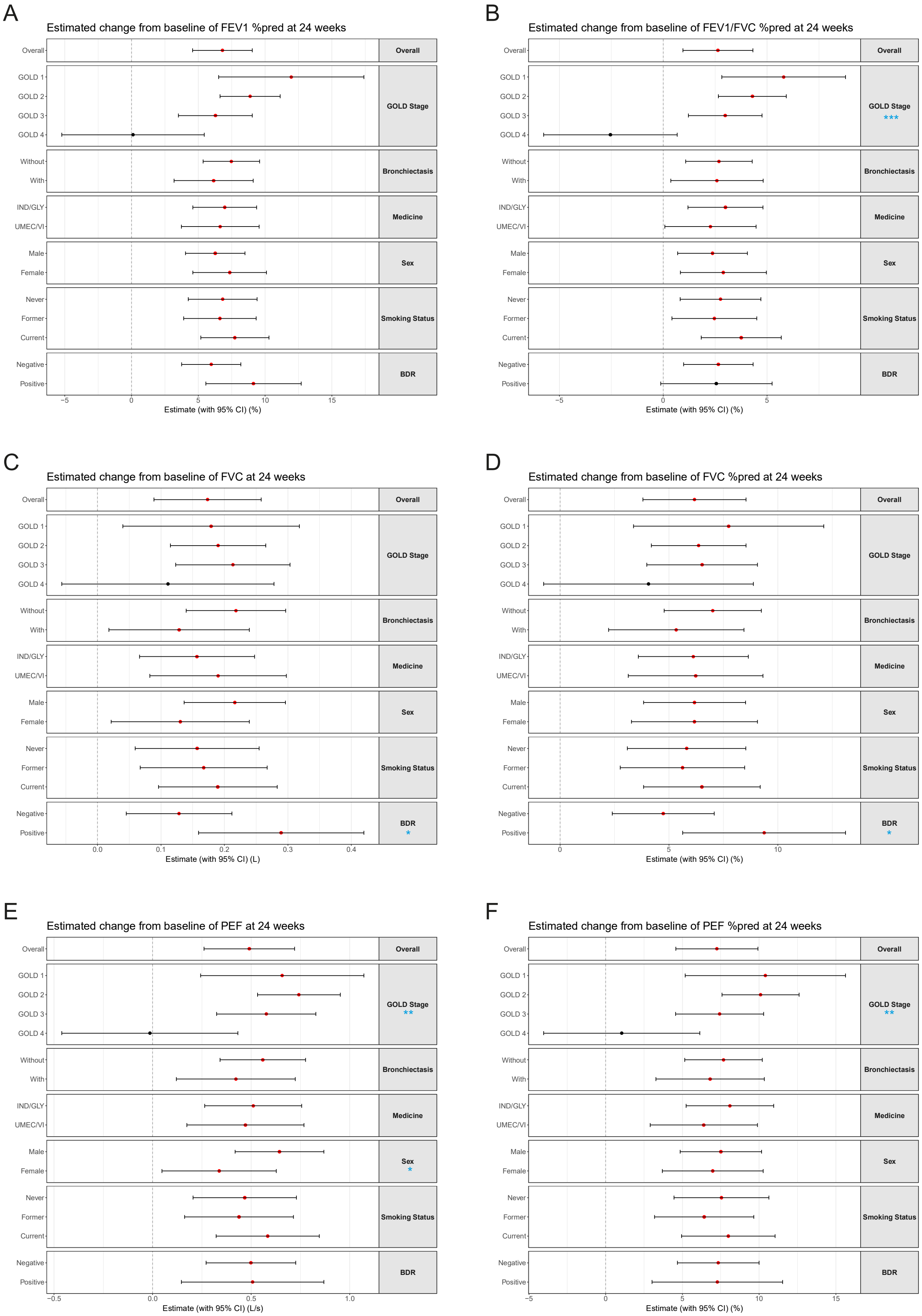
A
Estimated change from baseline of FEV1 %pred at 24 weeks
B
Estimated change from baseline of FEV1/FVC %pred at 24 weeks
C
Estimated change from baseline of FVC at 24 weeks
D
Estimated change from baseline of FVC %pred at 24 weeks
E
Estimated change from baseline of PEF at 24 weeks
F
Estimated change from baseline of PEF %pred at 24 weeks
Overall
GOLD 1
GOLD 2
GOLD 3
GOLD 4
GOLD Stage
Without
With
Bronchiectasis
IND/GLY
UMEC/VI
Medicine
Male
Female
Sex
Never
Former
Current
Smoking Status
Negative
Positive
BDR
Estimate (with 95% CI) (%)
Estimate (with 95% CI) (L)
Estimate (with 95% CI) (L/s)

G

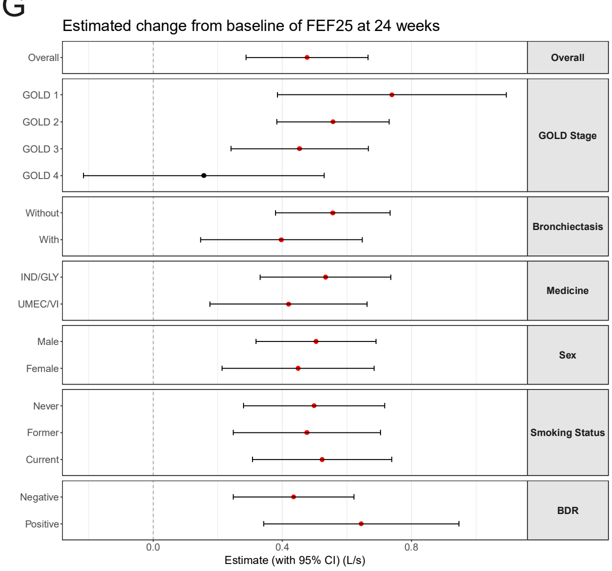
Estimated change from baseline of FEF25 at 24 weeks
Overall
GOLD 1
GOLD 2
GOLD 3
GOLD 4
GOLD Stage
Without
With
Bronchiectasis
IND/GLY
UMEC/VI
Medicine
Male
Female
Sex
Never
Former
Current
Smoking Status
Negative
Positive
BDR
0.0
0.4
0.8
Estimate (with 95% CI) (L/s)

H

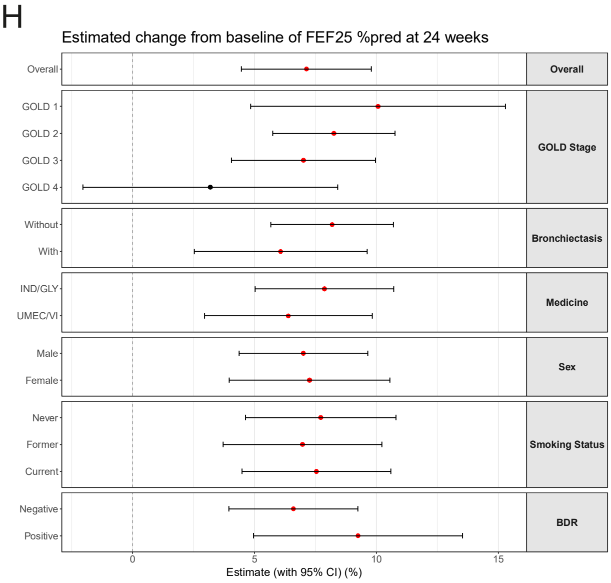
Estimated change from baseline of FEF25 %pred at 24 weeks
Overall
GOLD 1
GOLD 2
GOLD 3
GOLD 4
GOLD Stage
Without
With
Bronchiectasis
IND/GLY
UMEC/VI
Medicine
Male
Female
Sex
Never
Former
Current
Smoking Status
Negative
Positive
BDR
0
5
10
15
Estimate (with 95% CI) (%)

I

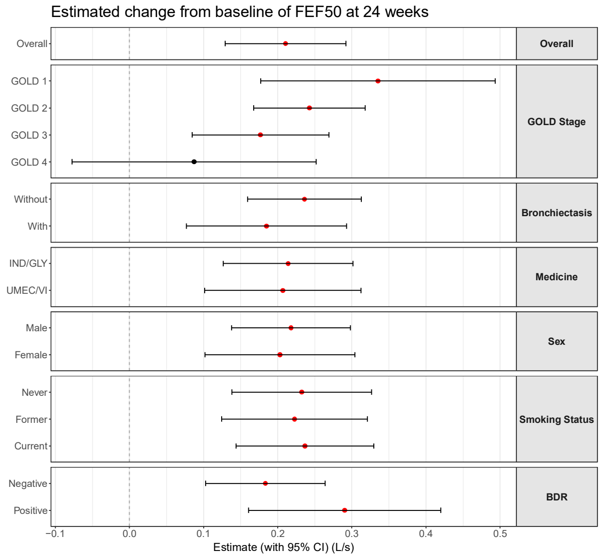
Estimated change from baseline of FEF50 at 24 weeks
Overall
GOLD 1
GOLD 2
GOLD 3
GOLD 4
GOLD Stage
Without
With
Bronchiectasis
IND/GLY
UMEC/VI
Medicine
Male
Female
Sex
Never
Former
Current
Smoking Status
Negative
Positive
BDR
−0.1
0.0
0.1
0.2
0.3
0.4
0.5
Estimate (with 95% CI) (L/s)

J

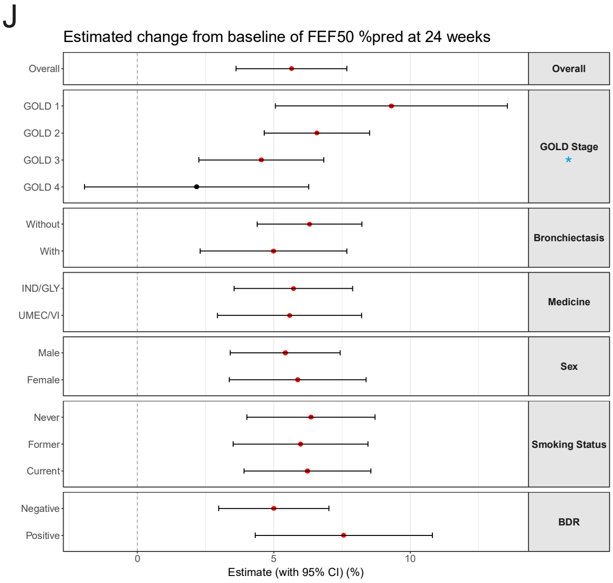
Estimated change from baseline of FEF50 %pred at 24 weeks
Overall
GOLD 1
GOLD 2
GOLD 3
GOLD 4
GOLD Stage
*
Without
With
Bronchiectasis
IND/GLY
UMEC/VI
Medicine
Male
Female
Sex
Never
Former
Current
Smoking Status
Negative
Positive
BDR
0
5
10
Estimate (with 95% CI) (%)

K

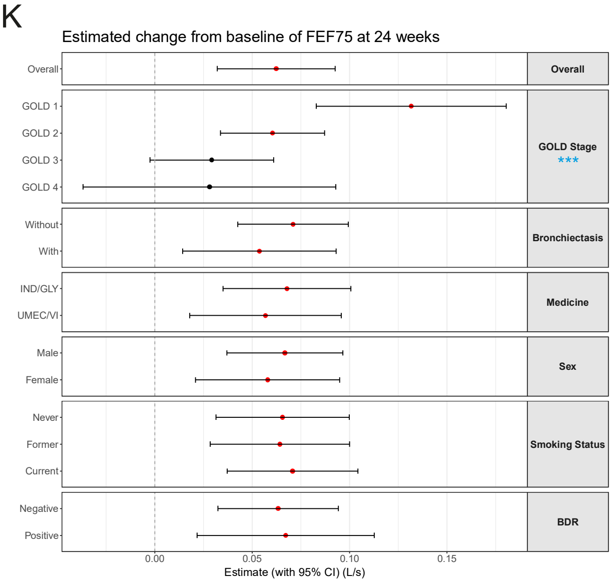
Estimated change from baseline of FEF75 at 24 weeks
Overall
GOLD 1
GOLD 2
GOLD 3
GOLD 4
GOLD Stage
***
Without
With
Bronchiectasis
IND/GLY
UMEC/VI
Medicine
Male
Female
Sex
Never
Former
Current
Smoking Status
Negative
Positive
BDR
0.00
0.05
0.10
0.15
Estimate (with 95% CI) (L/s)

L

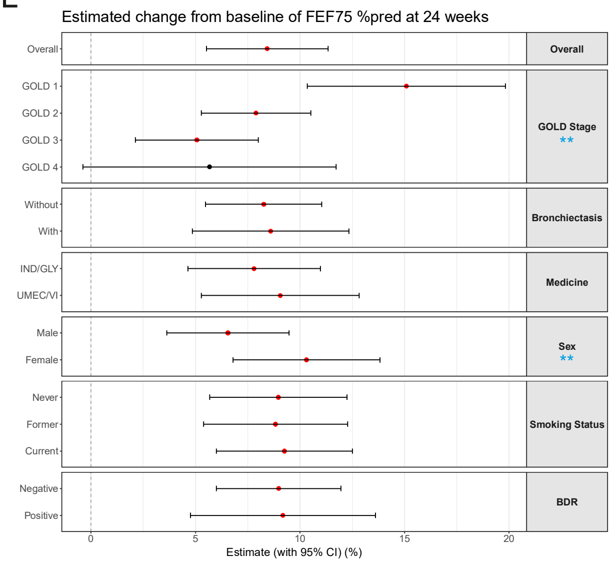
Estimated change from baseline of FEF75 %pred at 24 weeks
Overall
GOLD 1
GOLD 2
GOLD 3
GOLD 4
GOLD Stage
**
Without
With
Bronchiectasis
IND/GLY
UMEC/VI
Medicine
Male
Female
Sex
**
Never
Former
Current
Smoking Status
Negative
Positive
BDR
0
5
10
15
20
Estimate (with 95% CI) (%)

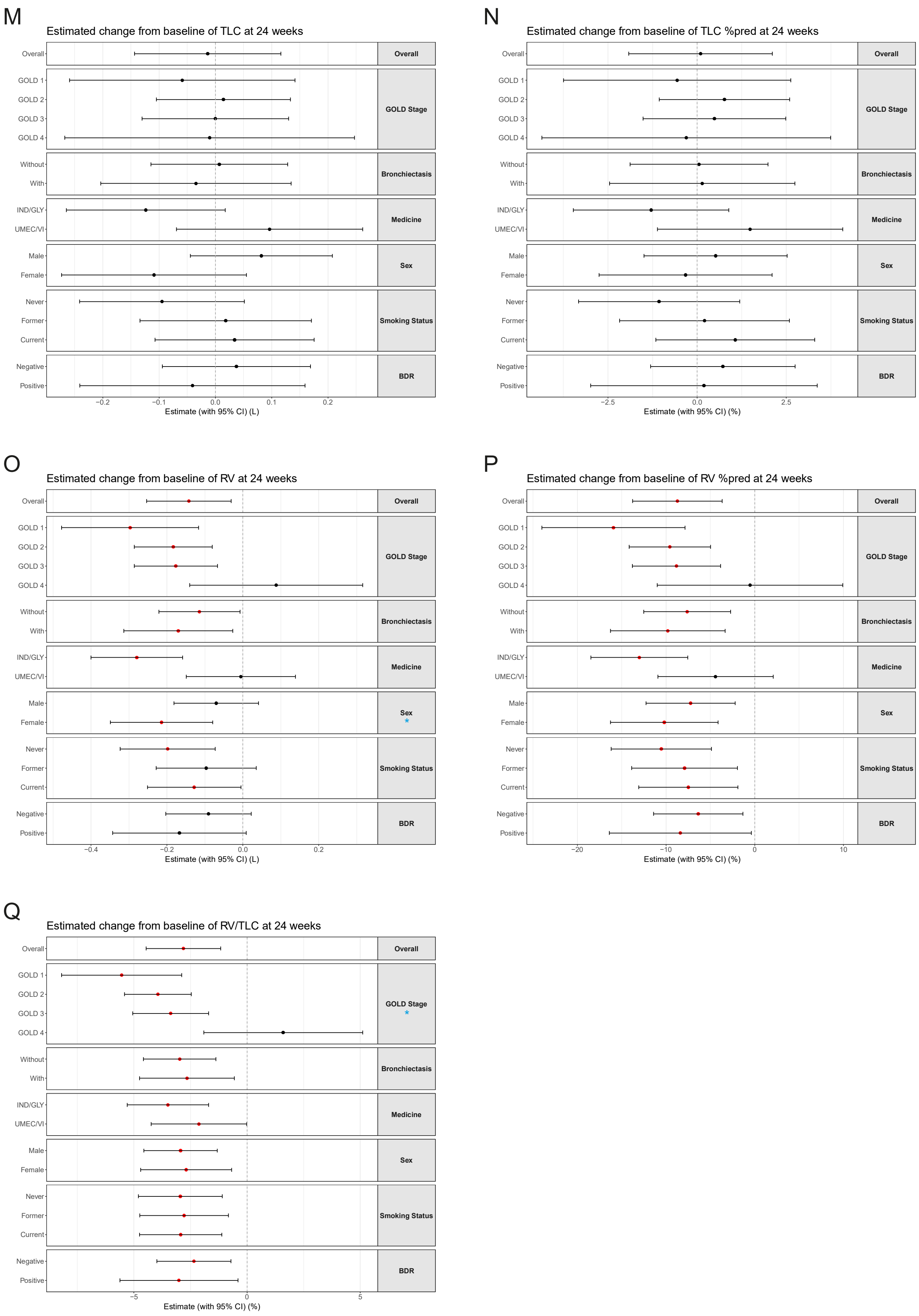
M
Estimated change from baseline of TLC at 24 weeks
Overall
GOLD 1
GOLD 2
GOLD 3
GOLD 4
Without
With
IND/GLY
UMEC/VI
Male
Female
Never
Former
Current
Negative
Positive
Overall
GOLD Stage
Bronchiectasis
Medicine
Sex
Smoking Status
BDR
−0.2 −0.1 0.0 0.1 0.2
Estimate (with 95% CI) (L)
N
Estimated change from baseline of TLC %pred at 24 weeks
Overall
GOLD 1
GOLD 2
GOLD 3
GOLD 4
Without
With
IND/GLY
UMEC/VI
Male
Female
Never
Former
Current
Negative
Positive
Overall
GOLD Stage
Bronchiectasis
Medicine
Sex
Smoking Status
BDR
−2.5 0.0 2.5
Estimate (with 95% CI) (%)
O
Estimated change from baseline of RV at 24 weeks
Overall
GOLD 1
GOLD 2
GOLD 3
GOLD 4
Without
With
IND/GLY
UMEC/VI
Male
Female
Never
Former
Current
Negative
Positive
Overall
GOLD Stage
Bronchiectasis
Medicine
Sex
*
Smoking Status
BDR
−0.4 −0.2 0.0 0.2
Estimate (with 95% CI) (L)
P
Estimated change from baseline of RV %pred at 24 weeks
Overall
GOLD 1
GOLD 2
GOLD 3
GOLD 4
Without
With
IND/GLY
UMEC/VI
Male
Female
Never
Former
Current
Negative
Positive
Overall
GOLD Stage
Bronchiectasis
Medicine
Sex
Smoking Status
BDR
−20 −10 0 10
Estimate (with 95% CI) (%)
Q
Estimated change from baseline of RV/TLC at 24 weeks
Overall
GOLD 1
GOLD 2
GOLD 3
GOLD 4
Without
With
IND/GLY
UMEC/VI
Male
Female
Never
Former
Current
Negative
Positive
Overall
GOLD Stage
*
Bronchiectasis
Medicine
Sex
Smoking Status
BDR
−5 0 5
Estimate (with 95% CI) (%)

R

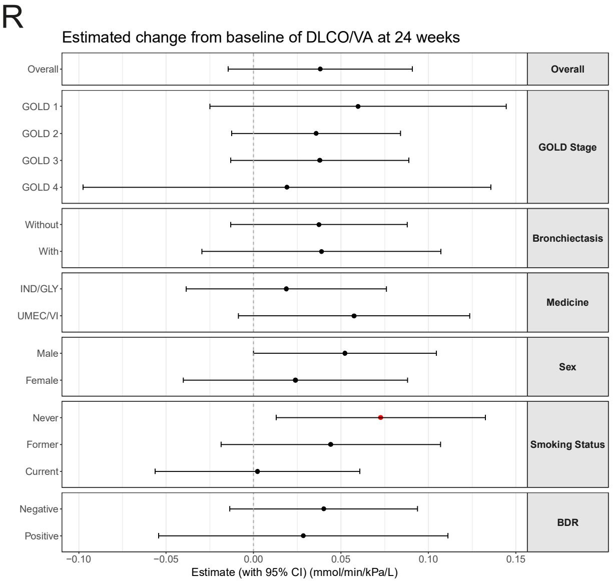

Estimated change from baseline of DLCO/VA at 24 weeks
Overall
GOLD 1
GOLD 2
GOLD 3
GOLD 4
GOLD Stage
Without
With
Bronchiectasis
IND/GLY
UMEC/VI
Medicine
Male
Female
Sex
Never
Former
Current
Smoking Status
Negative
Positive
BDR
−0.10
−0.05
0.00
0.05
0.10
0.15
Estimate (with 95% CI) (mmol/min/kPa/L)

S

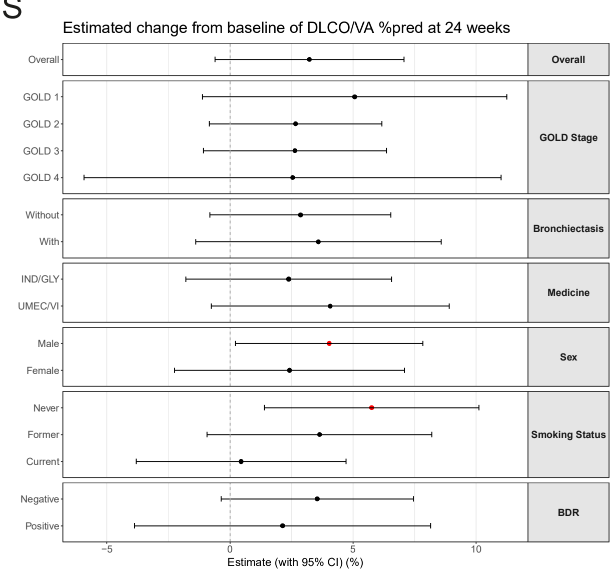

Estimated change from baseline of DLCO/VA %pred at 24 weeks
Overall
GOLD 1
GOLD 2
GOLD 3
GOLD 4
GOLD Stage
Without
With
Bronchiectasis
IND/GLY
UMEC/VI
Medicine
Male
Female
Sex
Never
Former
Current
Smoking Status
Negative
Positive
BDR
−5
0
5
10
Estimate (with 95% CI) (%)
