# Supplementary material for: Temporal and Partial Reversal of Airflow Limitation in Patients With COPD Treated With Single‐Inhaler Long‐Acting Dual Bronchodilators
Source: Clin Respir J. 2026 Apr 20;20(4):e70173. doi: 10.1111/crj.70173 (PMC13096688; doi:10.1111/crj.70173)
Supplement: Supplementary file 4 — Table S1: Baseline pulmonary function test result. [file CRJ-20-e70173-s008.docx]

**Supplementary table 1** Baseline pulmonary function test result

|  | **Entire cohort** | |  | **Initiators** | |
| --- | --- | --- | --- | --- | --- |
|  | **Number** | **Median (IQR)** |  | **Number** | **Median (IQR)** |
| FVC | 182 | 2.72 (0.89) |  | 143 | 2.70 (0.88) |
| FVC %pred | 182 | 76.20 (16.96) |  | 143 | 76.27 (18.55) |
| FEV1 | 182 | 1.51 (0.72) |  | 143 | 1.53 (0.78) |
| FEV1 %pred | 182 | 56.27 (21.20) |  | 143 | 56.30 (21.87) |
| FEV1/FVC | 182 | 57.52 (14.90) |  | 143 | 57.56 (13.30) |
| FEV1/FVC %pred | 182 | 74.10 (20.01) |  | 143 | 74.07 (17.97) |
| TLC | 174 | 5.10 (1.14) |  | 138 | 5.12 (1.12) |
| TLC %pred | 174 | 87.47 (13.71) |  | 138 | 87.07 (14.01) |
| RV | 174 | 2.38 (0.73) |  | 138 | 2.35 (0.70) |
| RV %pred | 174 | 115.19 (34.62) |  | 138 | 115.19 (34.03) |
| RV/TLC | 174 | 48.55 (10.86) |  | 138 | 48.55 (11.48) |
| PEF | 182 | 4.24 (1.99) |  | 143 | 4.28 (2.04) |
| PEF %pred | 182 | 48.98 (21.12) |  | 143 | 49.15 (22.64) |
| FEF25 | 182 | 1.90 (1.64) |  | 143 | 1.93 (1.81) |
| FEF25 %pred | 182 | 26.76 (22.55) |  | 143 | 26.85 (22.28) |
| FEF50 | 182 | 0.83 (0.52) |  | 143 | 0.85 (0.52) |
| FEF50 %pred | 182 | 21.41 (14.08) |  | 143 | 21.65 (13.90) |
| FEF75 | 178 | 0.26 (0.15) |  | 140 | 0.26 (0.16) |
| FEF75 %pred | 178 | 27.46 (15.27) |  | 140 | 27.32 (15.02) |
| DLCO/VA | 173 | 1.02 (0.46) |  | 137 | 1.05 (0.47) |
| DLCO/VA %pred | 173 | 73.13 (30.55) |  | 137 | 73.98 (30.52) |
| FeNO | 63 | 18.00 (11.00) |  | 51 | 18.00 (11.00) |

**Abbreviations:** IQR: interquartile range; DLCO/VA: Diffusing Capacity per Unit Alveolar Volume; FEF: Forced Expiratory Flow; FEV1: Forced Expiratory Volume in 1 second; FEV1/FVC: Ratio of Forced Expiratory Volume in 1s to Forced Vital Capacity; FVC: Forced Vital Capacity; PEF: Peak Expiratory Flow; RV: Residual Volume; RV/TLC: Residual Volume to Total Lung Capacity Ratio; TLC: Total Lung Capacity; %pred: percent predicted; FeNO: Fractional exhaled Nitric Oxide.
