# Supplementary material for: Temporal and Partial Reversal of Airflow Limitation in Patients With COPD Treated With Single‐Inhaler Long‐Acting Dual Bronchodilators
Source: Clin Respir J. 2026 Apr 20;20(4):e70173. doi: 10.1111/crj.70173 (PMC13096688; doi:10.1111/crj.70173)
Supplement: Supplementary file 5 — Table S2: Estimated change of spirometry parameters at 24 weeks and 1 year in initiators. [file CRJ-20-e70173-s006.docx]

**Supplementary table 2** Estimated change of spirometry parameters at 24 weeks and 1 year in initiators

| **Parameter** | **24 weeks** | | **1 year** | |
| --- | --- | --- | --- | --- |
|  | **Change [95% CI]** | ***P*-value^†^** | **Change [95% CI]** | ***P*-value^†^** |
| FVC | 0.219 L, [0.123, 0.316] | **<0.001** | 0.035 L, [-0.063, 0.133] | 0.478 |
| FVC %pred | 7.565 %, [4.884, 10.246] | **<0.001** | 2.651 %, [-0.102, 5.405] | 0.059 |
| FEV1 | 0.197 L, [0.125, 0.269] | **<0.001** | 0.102 L, [0.028, 0.175] | **0.007** |
| FEV1 %pred | 8.221 %, [5.721, 10.72] | **<0.001** | 5.418 %, [2.832, 8.004] | **<0.001** |
| FEV1/FVC | 2.441 %, [1.043, 3.838] | **0.001** | 2.711 %, [1.264, 4.159] | **<0.001** |
| FEV1/FVC %pred | 3.345 %, [1.43, 5.26] | **0.001** | 4.075 %, [2.091, 6.059] | **<0.001** |
| TLC | 0.057 L, [-0.096, 0.21] | 0.461 | -0.074 L, [-0.234, 0.086] | 0.361 |
| TLC %pred | 1.211 %, [-1.092, 3.514] | 0.301 | 0.264 %, [-2.16, 2.688] | 0.830 |
| RV | -0.118 L, [-0.242, 0.006] | 0.062 | -0.099 L, [-0.23, 0.032] | 0.140 |
| RV %pred | -9.052 %, [-14.551, -3.554] | **0.001** | -4.056 %, [-9.846, 1.734] | 0.169 |
| RV/TLC | -3.32 %, [-5.159, -1.481] | **<0.001** | -0.548 %, [-2.469, 1.372] | 0.574 |
| PEF | 0.565 L/s, [0.292, 0.837] | **<0.001** | 0.325 L/s, [0.046, 0.605] | **0.023** |
| PEF %pred | 8.336 %, [5.172, 11.5] | **<0.001** | 5.649 %, [2.363, 8.935] | **0.001** |
| FEF25 | 0.575 L/s, [0.363, 0.788] | **<0.001** | 0.407 L/s, [0.188, 0.626] | **<0.001** |
| FEF25 %pred | 8.284 %, [5.275, 11.293] | **<0.001** | 6.09 %, [2.974, 9.205] | **<0.001** |
| FEF50 | 0.254 L/s, [0.159, 0.348] | **<0.001** | 0.202 L/s, [0.105, 0.3] | **<0.001** |
| FEF50 %pred | 6.649 %, [4.305, 8.993] | **<0.001** | 5.58 %, [3.154, 8.007] | **<0.001** |
| FEF75 | 0.058 L/s, [0.022, 0.094] | **0.002** | 0.04 L/s, [0.003, 0.077] | **0.033** |
| FEF75 %pred | 8.186 %, [4.851, 11.522] | **<0.001** | 7.697 %, [4.274, 11.12] | **<0.001** |
| DLCO/VA | 0.008 mmol/min/kPa/L,  [-0.052, 0.069] | 0.790 | 0.002 mmol/min/kPa/L,  [-0.061, 0.065] | 0.957 |
| DLCO/VA %pred | 1.407 %, [-3.039, 5.852] | 0.533 | 0.466 %, [-4.169, 5.102] | 0.843 |

**Notes:** ^†^ The *P*-values were computed using the estimated marginal means (EMMs) obtained from the linear mixed-effects model, implemented via the R package “emmeans”. These *P*-values reflect the significance of the estimated means relative to a null hypothesis value of 0, with statistical significance defined as *P*<0.05. Values in bold indicate statistically significant results (*P*<0.05).

**Abbreviations:** CI: confidence interval; DLCO/VA: Diffusing Capacity per Unit Alveolar Volume; FEF: Forced Expiratory Flow; FEV1: Forced Expiratory Volume in 1 second; FEV1/FVC: Ratio of Forced Expiratory Volume in 1s to Forced Vital Capacity; FVC: Forced Vital Capacity; PEF: Peak Expiratory Flow; RV: Residual Volume; RV/TLC: Residual Volume to Total Lung Capacity Ratio; TLC: Total Lung Capacity; %pred: percent predicted.
