# Supplementary material for: Temporal and Partial Reversal of Airflow Limitation in Patients With COPD Treated With Single‐Inhaler Long‐Acting Dual Bronchodilators
Source: Clin Respir J. 2026 Apr 20;20(4):e70173. doi: 10.1111/crj.70173 (PMC13096688; doi:10.1111/crj.70173)
Supplement: Supplementary file 6 — Table S3: P‐value of fixed effects on spirometry parameters. [file CRJ-20-e70173-s002.docx]

**Supplementary table 3** *P*-value of fixed effects on spirometry parameters

|  | **Age** | **Smoking** | **Sex** | **Medicine** | **BE** | **BDR** | **GOLD** |
| --- | --- | --- | --- | --- | --- | --- | --- |
| FVC |  |  |  |  |  | * |  |
| FVC %pred |  |  |  |  |  | * |  |
| FEV1 |  |  |  |  |  |  |  |
| FEV1 %pred |  |  |  |  |  |  |  |
| FEV1/FVC |  |  |  |  |  |  | *** |
| FEV1/FVC %pred |  |  |  |  |  |  | *** |
| TLC | * |  |  |  |  |  |  |
| TLC %pred | * |  |  |  |  |  |  |
| RV |  |  | * |  |  |  |  |
| RV %pred | *** |  |  |  |  |  |  |
| RV/TLC |  |  |  |  |  |  | * |
| PEF |  |  | * |  |  |  | ** |
| PEF %pred |  |  |  |  |  |  | ** |
| FEF25 |  |  |  |  |  |  |  |
| FEF25 %pred |  |  |  |  |  |  |  |
| FEF50 |  |  |  |  |  |  |  |
| FEF50 %pred |  |  |  |  |  |  | * |
| FEF75 |  |  |  |  |  |  | *** |
| FEF75 %pred | *** |  | ** |  |  |  | ** |
| DLCO/VA |  |  |  |  |  |  |  |
| DLCO/VA %pred |  |  |  |  |  |  |  |

**Notes:** The table presents P-values indicating subgroup response differences derived from linear mixed-effects models (LMM). All demographic and clinical characteristics used for subgrouping were treated as fixed effects in the LMM analyses. P-values greater than 0.05 are presented as blank. *: P<0.05; **: P<0.01; ***: P<0.001.

**Abbreviations:** BE: Bronchiectasis; BDR: bronchodilator response; DLCO/VA: Diffusing Capacity per Unit Alveolar Volume; FEF: Forced Expiratory Flow; FEV1: Forced Expiratory Volume in 1 second; FEV1/FVC: Ratio of Forced Expiratory Volume in 1s to Forced Vital Capacity; FVC: Forced Vital Capacity; PEF: Peak Expiratory Flow; RV: Residual Volume; RV/TLC: Residual Volume to Total Lung Capacity Ratio; TLC: Total Lung Capacity; %pred: percent predicted.
