# Supplementary material for: Temporal and Partial Reversal of Airflow Limitation in Patients With COPD Treated With Single‐Inhaler Long‐Acting Dual Bronchodilators
Source: Clin Respir J. 2026 Apr 20;20(4):e70173. doi: 10.1111/crj.70173 (PMC13096688; doi:10.1111/crj.70173)
Supplement: Supplementary file 7 — Table S4: Baseline patient demographics and characteristics of reversed and non‐reversed group. [file CRJ-20-e70173-s007.docx]

**Supplementary table 4** Baseline patient demographics and characteristics of reversed and non-reversed group

| **Demographics and Characteristics** | | **Reversed**  **(N = 21)** | **Non-reversed**  **(N = 161)** | ***P*-value^†^** |
| --- | --- | --- | --- | --- |
| Sex | Male, N (%)  Female, N (%) | 17 (81)  4 (19) | 133 (82.6)  28 (17.4) | 1.000 |
| Age (year), mean ± SD |  | 69.5 ± 8.5 | 69.3 ± 8.9 | 0.944 |
| Smoking status | Current, N (%)  Former, N (%)  Never, N (%)  Unknown, N (%) | 4 (19)  10 (47.6)  5 (23.8)  2 (9.5) | 52 (32.3)  39 (24.2)  32 (19.9)  38 (23.6) | 0.084 |
| Comorbidities | Any of predetermined, N (%) | 15 (71.4) | 115 (71.4) | 1.000 |
| Bronchiectasis | With, N (%)  Without, N (%) | 1 (4.8)  20 (95.2) | 19 (11.8)  142 (88.2) | 0.549 |
| Treatment | IND/GLY, N (%)  UMEC/VI, N (%) | 15 (71.4)  6 (28.6) | 107 (66.5)  54 (33.5) | 0.835 |
| Baseline treatment | None, N (%)  Any prior treatment, N (%) | 15 (71.4)  6 (28.6) | 128 (79.5)  33 (20.5) | 0.572 |
| GOLD grade | 1, N (%)  2, N (%)  3, N (%)  4, N (%) | 3 (14.3)  18 (85.7)  0 (0)  0 (0) | 7 (4.3)  86 (53.4)  60 (37.3)  8 (5) | **0.001** |
| BDR | Negative, N (%)  Positive, N (%)  NA, N (%) | 11 (52.4)  2 (9.5)  8 (38.1) | 97 (60.2)  10 (6.2)  54 (33.5) | 0.735 |

**Notes:** ^†^ Differences between reversed and non-reversed group were calculated by either Student’s t-test or chi-square tests, as appropriate, presented in P-value. Values in bold indicate statistically significant results (P<0.05).

**Abbreviations:** SD: standard deviation; BDR: bronchodilator response; NA: not applicable.
