# Supplementary material for: Temporal and Partial Reversal of Airflow Limitation in Patients With COPD Treated With Single‐Inhaler Long‐Acting Dual Bronchodilators
Source: Clin Respir J. 2026 Apr 20;20(4):e70173. doi: 10.1111/crj.70173 (PMC13096688; doi:10.1111/crj.70173)
Supplement: Supplementary file 8 — Table S5: Odds ratios for various baseline lung function parameters, demographics, and characteristics in relation to the likelihood of FEV1/FVC reversal. [file CRJ-20-e70173-s003.docx]

**Supplementary table 5** Odds ratios for various baseline lung function parameters, demographics, and characteristics in relation to the likelihood of FEV1/FVC reversal

| **Baseline lung function** | **OR** | **95% CI** | **Center^†^** | **Scale^†^** | ***P*-value** |
| --- | --- | --- | --- | --- | --- |
| FVC | 1.23 | [0.78, 1.94] | 2.72 | 0.72 | 0.360 |
| FVC %pred | 1.15 | [0.73, 1.8] | 76.31 | 15.14 | 0.531 |
| FEV1 | **2.19** | [1.4, 3.53] | 1.52 | 0.52 | **0.001** |
| FEV1 %pred | **2.65** | [1.61, 4.64] | 55.17 | 15.45 | **<0.001** |
| FEV1/FVC | **51.68** | [11.17, 409.37] | 55.63 | 9.41 | **<0.001** |
| FEV1/FVC %pred | **13.22** | [4.78, 48.84] | 72.26 | 12.7 | **<0.001** |
| TLC | 0.74 | [0.45, 1.22] | 5.07 | 0.93 | 0.245 |
| TLC %pred | **0.62** | [0.35, 1.05] | 87.61 | 11.32 | **0.090** |
| RV | **0.39** | [0.19, 0.73] | 2.45 | 0.63 | **0.006** |
| RV %pred | **0.37** | [0.17, 0.72] | 118.2 | 30.28 | **0.007** |
| RV/TLC | **0.46** | [0.26, 0.78] | 48.46 | 8.28 | **0.006** |
| PEF | **3.22** | [1.99, 5.57] | 4.41 | 1.63 | **<0.001** |
| PEF %pred | **4.01** | [2.36, 7.48] | 51.09 | 16.5 | **<0.001** |
| FEF25 | **3.66** | [2.29, 6.36] | 2.18 | 1.27 | **<0.001** |
| FEF25 %pred | **4.3** | [2.57, 7.99] | 29.86 | 16.64 | **<0.001** |
| FEF50 | **2.87** | [1.85, 4.7] | 0.9 | 0.45 | **<0.001** |
| FEF50 %pred | **3.54** | [2.17, 6.24] | 22.71 | 10.46 | **<0.001** |
| FEF75 | **1.68** | [1.12, 2.54] | 0.29 | 0.12 | **0.011** |
| FEF75 %pred | 1.14 | [0.72, 1.62] | 29.59 | 20.15 | 0.473 |
| DLCO/VA | **2.26** | [1.35, 3.99] | 1.02 | 0.34 | **0.003** |
| DLCO/VA %pred | **2.4** | [1.39, 4.42] | 73.48 | 23.26 | **0.003** |
| **Demographics and Characteristics** | **OR** | **95% CI** | **Center** | **Scale** | ***P*-value** |
| Sex | 0.89 | [0.3, 3.29] | - | - | 0.851 |
| Age | 1 | [0.95, 1.06] | - | - | 0.945 |
| Smoking | 3.33 | [1.03, 12.88] | - | - | 0.055 |
| Bronchiectasis | 0.37 | [0.02, 1.96] | - | - | 0.350 |
| Treatment | 0.79 | [0.27, 2.07] | - | - | 0.649 |
| Baseline Treatment | 1.55 | [0.52, 4.15] | - | - | 0.399 |
| GOLD grade | 0.49 | [0.12, 2.43] | - | - | 0.331 |
| BDR | 0.77 | [0.29, 2.09] | - | - | 0.589 |

**Notes:** ^†^ Baseline lung function parameters were scaled for improved comparability using the “scale” function in R, where “center” and “scale” represent the mean and standard deviation, respectively. The *P*-value was derived from a logistic regression analysis performed on the scaled data. Values in bold indicate statistically significant results (*P*<0.05).

**Abbreviations:** OR: odds ratio; CI: confidence interval; BDR: bronchodilator response. DLCO/VA: Diffusing Capacity per Unit Alveolar Volume; FEF: Forced Expiratory Flow; FEV1: Forced Expiratory Volume in 1 second; FEV1/FVC: Ratio of Forced Expiratory Volume in 1s to Forced Vital Capacity; FVC: Forced Vital Capacity; PEF: Peak Expiratory Flow; RV: Residual Volume; RV/TLC: Residual Volume to Total Lung Capacity Ratio; TLC: Total Lung Capacity; %pred: percent predicted.
